# Transcriptome-Wide Analysis of Pituitary and Ectopic Adrenocorticotropic Hormone-Secreting Tumors

**DOI:** 10.3390/cancers17040658

**Published:** 2025-02-15

**Authors:** Anton A. Buzdin, Rustam N. Heydarov, Olga O. Golounina, Maria V. Suntsova, Alina V. Matrosova, Ekaterina V. Bondarenko, Sergey A. Roumiantsev, Maksim I. Sorokin, Roman V. Kholodenko, Irina V. Kholodenko, Vladimir P. Chekhonin, Evgeniya V. Plaksina, Liudmila Y. Rozhinskaya, Galina A. Melnichenko, Zhanna E. Belaya

**Affiliations:** 1 Endocrinology Research Center, Moscow 117036, Russia; rustam.heydarov@gmail.com (R.N.H.); golounina.olga@endocrincentr.ru (O.O.G.); suntsova_m_v@staff.sechenov.ru (M.V.S.); matrosova.alina@endocrincentr.ru (A.V.M.); bondarenko.ekaterina@endocrincentr.ru (E.V.B.); rumyantsev.sergey@endocrincentr.ru (S.A.R.); sorokin@oncobox.com (M.I.S.); chehonin.vladimir@endocrincentr.ru (V.P.C.); plaksina.evgenia@endocrincentr.ru (E.V.P.); rozhinskaya.ludmila@endocrincentr.ru (L.Y.R.); melnichenko.galina@endocrincentr.ru (G.A.M.); jannabelaya@gmail.com (Z.E.B.); 2 Institute of Personalized Oncology, I.M. Sechenov First Moscow State Medical University, Moscow 119991, Russia; 3 Moscow Institute of Physics and Technology, Dolgoprudny 141701, Russia; 4 Shemyakin-Ovchinnikov Institute of Bioorganic Chemistry, Russian Academy of Sciences, Moscow 117997, Russia; khol@mail.ru; 5 Oncobox LLC, Moscow 119991, Russia; 6 Laboratory of Cell Biology, Orekhovich Institute of Biomedical Chemistry, Moscow 119121, Russia; irkhol@yandex.ru

**Keywords:** neuroendocrine tumors, adrenocorticotropic hormone (ACTH), Cushing’s syndrome, POMC, ectopic ACTH syndrome, pituitary, RNA sequencing, transcriptomics, molecular diagnostics, emerging therapeutic targets

## Abstract

**Background/Objectives:** Endogenous Cushing’s syndrome (CS) is a rare neuroendocrine disorder often resulting from adrenocorticotropic hormone (ACTH) production by a pituitary or ectopic tumor. The biological mechanisms underlying ACTH-secreting tumors, especially non-pituitary, remain poorly understood. This study aimed to explore the transcriptomic profiles of pituitary and ectopic ACTH-secreting tumors (ASTs) to identify potential biomarkers for differential diagnosis and new therapeutic targets. **Methods:** In this study, we performed a transcriptome-wide analysis of twenty-pituitary, one pancreatic, and six lung ASTs, along with seven pituitary, eight pancreatic, and eight lung healthy control tissue samples, respectively. **Results:** The transcriptomic analysis revealed distinct profiles between pituitary and ectopic ACTH-secreting tumors. In all ASTs, except for the pancreatic sample, we found an overexpression of the TBX19 and PITX1 transcription factor genes, which promote the overexpression of POMC. The transcriptional profiles of ectopic lung ASTs showed marked overexpression of the gastrin-releasing peptide and calcitonin genes, which may be potentially useful for diagnostic purposes. We identified several transcriptionally activated molecular pathways in ASTs that might be targetable with existing medications to treat cancer, the most potentially promising of which are RET inhibitors and an IL6R inhibitor. We also predicted and experimentally validated that corticotropinomas express ganglioside 2 molecules, offering new therapeutic targets for Cushing’s disease. **Conclusions:** This study provides new insights into the molecular features of ACTH-secreting tumors and identifies potential diagnostic markers and therapeutic targets. Distinct transcriptomic profiles in pituitary and ectopic tumors highlight unique biomarkers that may improve diagnostic accuracy and facilitate the development of targeted therapies for ACTH-dependent CS.

## Simple Summary

Endogenous Cushing’s syndrome is a rare disorder caused by excessive cortisol most commonly due to elevated ACTH produced by neuroendocrine tumors. These tumors most commonly arise in the pituitary gland (Cushing’s disease) but can also originate in other parts of the body (ectopic ACTH syndrome). Distinguishing between pituitary and ectopic causes is essential for choosing the best treatment, yet current tools are not always optimal. Our research compares the transcriptome patterns of pituitary and ectopic tumors to discover unique markers and possible new drug targets. This research provides new insights into specific molecules and pathways linked to each tumor type, common mechanisms of ACTH-secretion, and suggests promising diagnostics and treatment targets for future research. The analysis of the experimental efficacy of existing medications registered for other oncological disorders may provide new perspectives for these rare orphan disorders in cases where traditional treatment options have been exhausted.

## 1. Introduction

Endogenous Cushing’s syndrome (CS) is a rare neuroendocrine disorder characterized by either secondary cortisol increases due to an adrenocorticotropic hormone (ACTH)-secreting pituitary tumor (Cushing’s disease (CD)), an ACTH-secreting neuroendocrine tumor (NET) of non-pituitary origin (ectopic ACTH syndrome (EAS)), or by the primarily adrenal autonomous overproduction of cortisol [1]. Clinical complications of CS include arterial hypertension, hypokalemia, increased probability of heart failure and major cardiovascular events, diabetes mellitus, infections, osteoporosis with low-traumatic fractures, and severe psychiatric disorders [2,3]. CS of any origin is associated with increased disability and mortality [4,5]. ACTH-dependent CS presents significant challenges in differential diagnostics, tumor visualization, and subsequent treatment [1]. An ACTH-producing pituitary neuroendocrine tumor (PitNET) is not visualized on MRI with gadolinium in 20% of cases [6]. Conversely, up to 20% of the healthy population carry a non-secreting pituitary incidentaloma, which may coexist with an ACTH-producing neuroendocrine tumor (NET) of any localization, making the differential diagnostics even more challenging [7]. ACTH secretion and the loss of the negative cortisol–ACTH loop in patients with CD are the subjects of active research, in addition to cell cycle alteration and pituitary tumor growth [8]. The ability of non-pituitary ACTH-secreting tumors to secrete ACTH is even less well understood. EAS is predominantly caused by lung, mediastinal, pancreatic ACTH-secreting tumors, and more rarely, by medullary thyroid cancer, pheochromocytoma, or any other rare tumor transformation of metastatic prostate or breast cancers [9]. ACTH-secreting tumors are usually small and might remain occult for many years in up to 20% of cases [10,11], which emphasizes the necessity for tissue-specific biomarkers and/or treatment targeting ACTH production.

The molecular biology of ACTH-secreting tumors remains poorly understood, largely due to their rarity and heterogeneity. Previous research has primarily focused on pituitary ACTH-secreting adenomas, revealing alterations in transcription factors such as Tpit (also known as TBX19) and Pitx1, which regulate the expression of proopiomelanocortin (POMC), the precursor molecule for ACTH [12,13,14]. These transcription factors are essential for the differentiation and function of corticotroph cells in the pituitary gland. However, the molecular mechanisms underlying ectopic ACTH secretion remain poorly characterized, in part due to the diverse tissue origins of these tumors and the limited number of cases available for study [15].

The application of transcriptome-wide analyses, particularly RNA sequencing (RNA-seq), offers a powerful approach to comprehensively profile gene expression patterns in tumors. RNA-seq enables the unbiased detection of all transcribed genes, including novel transcripts and splice variants, providing insights into the complex molecular networks driving tumor behavior. This technology has been successfully utilized to identify diagnostic markers, prognostic indicators, and therapeutic targets in various cancers [16]. For rare tumors like ACTH-secreting tumors, transcriptome-wide studies are especially valuable, as they can uncover molecular signatures that may not be apparent through targeted analyses [17].

In this context, our study aims to fill the existing knowledge gap by performing a comprehensive transcriptome-wide comparison of gene expression profiles in pituitary and ectopic ACTH-secreting tumors. We conducted RNA sequencing analyses on samples from pituitary ACTH-secreting tumors, ectopic ACTH-secreting tumors isolated from the lung and pancreas, along with healthy pituitary, lung, and pancreatic tissues. By examining these gene expression profiles, we sought to identify the common and unique molecular features of these tumors to elucidate the underlying biology of ectopic ACTH-secreting tumors, discover potential diagnostic biomarkers, and explore new therapeutic avenues that could improve the management of patients with Cushing’s syndrome due to ACTH hypersecretion.

## 2. Materials and Methods

### 2.1. Ethical Considerations

This study was conducted in accordance with the 1964 Declaration of Helsinki and was approved by the Local Ethics Committee of the Endocrinology Research Center (approval number 12) on 29 June 2022 for studies involving human cancer tissue biopsy materials. Written informed consents were obtained from all subjects involved in this study or from their legal representatives to perform this study and to publish its results in the form of a scientific paper, including the option of publishing depersonalized RNA sequencing data.

### 2.2. Clinical Data

The diagnosis of ACTH-dependent endogenous hypercortisolism was based on the following laboratory tests: increased 24-h urinary free cortisol (24hUFC) level; late-night salivary cortisol and/or serum cortisol at 23:00 and/or unsuppressed serum cortisol after a 1 mg overnight dexamethasone suppression test (cut-off point 50 nmol/L), along with a morning ACTH level ≥ 10 pg/mL. The 24hUFC (100–379 nmol/L) level was measured using Vitros Eci (QuidelOrtho, San Diego, CA, USA). The plasma levels of ACTH (reference range: morning 7.2–63.3 pg/mL, late-night 2–25.5 pg/mL), late-night serum cortisol (64–327 nmol/L), and late-night salivary cortisol (0.5–9.6 nmol/L) were measured via ECLIA on a Cobas e60 (Roche, Rotkreuz, Switzerland).

Bilateral inferior petrosal sinus sampling (BIPSS) with a stimulating agent (Desmopressin Acetate 8 mcg) was used to differentiate within ACTH-dependent hypercortisolism. EAS was established when the central-to-peripheral gradient of ACTH was below 2 before stimulation and below 3 after stimulation. Various imaging techniques such as pituitary magnetic resonance imaging (MRI) with gadolinium, spiral thin-slice computed tomography (CT) of the cervical–thoracic–abdominal regions, octreoscan using ^99m^Tc-tectrotide, and/or positron emission tomography/computed tomography (PET/CT) using ^68^Ga-DOTA-68-labeled somatostatin receptor ligands were performed to locate the ACTH-producing tumor. Only tissue samples with a clear histopathological diagnosis for each localization of the primary tumor were enrolled in this study. ACTH production was confirmed using an immunohistochemical method for the expression of ACTH.

### 2.3. Tumor Biosamples and RNA Sequencing

This study involved tumor tissue samples obtained from 36 patients who underwent surgery at the Endocrinology Research Center in Moscow, Russia. The biosamples were formalin-fixed, paraffin-embedded (FFPE) tumor tissue samples with at least 60% tumor cells, as evaluated by a pathologist. Tumoral tissue specimens of the pituitary were obtained during transsphenoidal surgery. Corticotroph tumors were characterized via immunostaining for ACTH. It should be noted that transcription factor TPIT staining was not performed for the identification of pituitary ACTH-secreting tumors in this study. However, tumor identification relied on established diagnostic criteria, including clinical features, biochemical assessments, imaging results, and ACTH immunostaining. While these methods are considered robust for diagnosing ACTH-secreting pituitary tumors, the inclusion of TPIT staining in future studies could provide additional validation for tumor subtype characterization. The sampling included twenty-nine biosamples of ACTH-secreting pituitary adenomas (pituitary ACTH-secreting tumors), six biosamples of lung carcinoids with ectopic ACTH secretion (lung ACTH-secreting tumors), and one case of a pancreatic neuroendocrine tumor with ectopic ACTH secretion (pancreatic ACTH-secreting tumor). Reference pituitary tissues comprised seven samples of pathologically normal pituitary tissue obtained during the exploratory neurosurgery of non-visualized (*n* = 4) and 3 mm pituitary corticotropinoma (*n* = 3). In 5 of these cases, remission was achieved after the exploratory neurosurgery; this means that the corticotropinomas were lost in vacuum tubes, and normal pituitary samples were sent for histological evaluation. In two cases, remission was not achieved, but healthy pituitary samples were confirmed through histologic evaluation. None of these patients had hypopituitarism with the exception of the expected secondary adrenal insufficiency after the neurosurgery. The diagnoses, age, and gender details of the individuals involved are given in Supplementary Table S1.

Reference healthy lung and healthy pancreas biosamples were obtained from the ANTE database of RNA sequencing profiles, which we had previously established for patients killed in road accidents [18] and obtained using the same reagents, protocols, and software as in the present study.

For every patient’s biosample, written informed consent to participate in this study was obtained from the patient or his/her legal representative. The consent procedure and the design of the study were guided and approved by the Local Ethics Committee of the Endocrinology Research Center (approval number 12), Moscow, Russia, with the date of approval being 29 June 2022.

RNA extraction, library preparation, sequencing, and RNA-seq data processing were performed as previously described [18]. RNA was extracted from FFPE samples using the HiPure FFPE RNA Kit (Magen, Guangdong, China). Sequencing libraries were prepared using the KAPA RNA HyperPrep Kit with RiboErase (Roche, Rotkreuz, Switzerland). NGS was performed on a NovaSeq 6000 (Illumina, San Diego, CA, USA) engine in paired-end mode, with 100 bp read lengths. Raw sequencing data were demultiplexed and converted into the FASTQ format using the bcl2fastq version 2.17 software (Illumina).

### 2.4. RNA Sequencing Data Processing

The protocol for processing RNA sequencing data involved several steps. Initially, the quality of the sequencing reads was assessed using FastQC (version 0.12.1). The RNA-seq FASTQ files were then processed using the STAR aligner (version 2.7.2b) in “GeneCounts” mode, with the Ensembl human transcriptome annotation (GRCh38 build, transcript annotation GRCh38.89) as the reference. Finally, for further assessments, only the samples with high-quality experimental RNA sequencing profiles reaching the threshold of 3.5 × 10^6^ gene-mapped reads were selected [18].

### 2.5. Principal Component Analysis

Principal component analysis (PCA) was conducted on normalized gene expression data using the plotPCA function from the DESeq2 (version 1.46.0) package in R (version 4.4.0). Before performing PCA, the read count data were normalized and transformed using the variance-stabilizing transformation (VST). This transformation helps make the data distribution more symmetrical in order to enhance the quality of the subsequent analysis.

### 2.6. Detection of Fusion Transcripts

Fusion transcripts were identified using the STAR-Fusion software [19] (version STAR-2.7.2b) within the RNA sequencing profiles. The pre-assembled genome build GRCh38_gencode_v33_CTAT_lib_Apr062020.plug-n-play was used as the reference genome sequence. Default parameters were used for STAR-Fusion. Fusion candidate files were generated, followed by the extraction of relevant associated RNA sequencing reads.

Fusions where the JunctionReadCount (number of reads containing the fusion junction) and SpanningFragCount (number of reads flanking the fusion region) did not exceed 2 were removed to exclude low-confidence candidates. Additionally, fusions involving mitochondrial genes were discarded.

The resulting data were then manually inspected using UCSC BLAT and the UCSC Genome Browser (https://genome-euro.ucsc.edu, accessed on 1 April 2024). The evaluation of fusion candidates was conducted based on the following criteria: (i) whether the sequencing read spans an exon junction between two distinct, previously characterized transcripts, (ii) whether the junction site precisely corresponds to exon boundaries of known protein-coding genes, taking into account established splice sites, and (iii) whether both transcripts within the sequencing read are aligned in the same orientation, indicating the presence of a putative fusion RNA. Reads that met these criteria were considered as supporting evidence for the gene fusion transcripts.

### 2.7. Analysis of Differential Gene Expression

The analysis of differential gene expression was conducted using the DESeq2 package (version 1.46.0) in R (version 4.4.0). The initial data utilized were the read counts at the gene level. Genes with low read counts were filtered out before starting the analysis. The significance of differential expression was determined based on the Benjamani–Hochberg adjusted *p*-value threshold, which was set at no more than 0.05. Additionally, a threshold for the absolute value of the logarithm of the fold change in expression (log fold change, log_2_FC) was set at 2.

### 2.8. Gene Ontology Enrichment Analysis

Gene ontology (GO) enrichment analysis was performed using the clusterProfiler package (version 4.14.4) in R (version 4.4.0). The initial data consisted of lists of differentially expressed genes (DEGs). The enrichGO function was applied to identify significantly enriched GO terms in the categories of biological processes, cellular components, and molecular functions. All genes that were tested in the differential expression analysis were used as the background gene set. The enrichment results were visualized using the dotplot function from the clusterProfiler package.

### 2.9. Calculation of Pathway Activation Levels

The pathway activation level (PAL) is an integral quantitative and qualitative characteristic that reflects changes in the expression levels of genes participating in a molecular pathway. PAL values were calculated as follows:

$${PAL}_{p}=100\times \sum_{n}{ARR}_{np}\times lg({CNR}_{n})/\sum_{n}{|ARR}_{np}|$$

where PALp represents the PAL for a pathway *p*, and CNRn is the case-to-normal ratio for a gene *n*. The ARR (activator/repressor role) is a value that depends on the function of the gene product in pathway *p*. ARRnp is defined as follows: a value of −1 when the product of gene *n* inhibits pathway *p*; a value of 1 when the product of gene *n* activates pathway *p*; and 0 when the role of gene *n* in pathway *p* is ambiguous. The CNRn value is calculated as the ratio of a quantitative metric level for gene *n* in the biosample under study to the average level for gene *n* in the control group [20]. For PAL calculations and visualization, the OncoboxPD database and software (https://open.oncobox.com, accessed on 12 June 2024) were used [21].

### 2.10. Simulation of Targeted Drug Efficacy

The drug score, also known as the balanced efficiency score (BES), for targeted cancer drugs was calculated using the Oncobox method for RNA sequencing gene expression data based on the analysis of targeted molecular pathway activation and the relative expression levels of drug target genes, as detailed in a previously published study [22]. Similarly, for therapeutics targeting ganglioside GD2, a 2-gene sensitivity signature was calculated using RNA expression data, following the methodologies outlined in earlier research [23].

### 2.11. Testing of Intersection Significance

To determine whether the observed number of overlapping differentially expressed genes or pathways between intersecting datasets is significant, 10,000 random intersections were performed by randomly permuting gene names and intersecting random gene groups of the same sizes for each comparison. The *p*-value for the significance of the intersection was calculated as the proportion of random intersections that were equal to or greater than the number of overlapping genes observed experimentally.

### 2.12. Isolation of Tumor Cells from Biopsies of Patients with Pituitary ACTH-Secreting Tumors

The tissue sample was delivered to the laboratory within 3 h after surgery, preserved in PBS with an antibiotic/antimycotic solution (Gibco, Grand Island, NE, USA), and maintained at +4 °C. The tube with the tumor tissue was centrifuged for 5 min at 1200 rpm, and the supernatant was discarded. A 0.35% type I collagenase solution (Gibco, USA) was added to the tissue; large pieces of the tissue were additionally minced with scissors and incubated in the enzyme for 1 h at 37 °C. Following incubation, the tissue was thoroughly suspended to enhance disaggregation and then centrifuged for 5 min at 300 g. The pellet was resuspended in a complete growth medium comprising DMEM/F12 supplemented with 20% FBS, penicillin/streptomycin, and L-glutamine (Gibco, USA). The cell suspension was plated into wells of a 6-well plate pre-coated with laminin-521 (STEMCELL Technologies, Kent, WA, USA). The medium was changed 24 h after isolation. Cell morphology was assessed using a Zeiss Axiovert 40CFL phase-contrast microscope (Carl Zeiss, Jena, Germany), and cells were photographed using a Nikon D5000 camera (Nikon, Tokyo, Japan).

### 2.13. Staining of Cells with Antibodies to Surface Marker Molecules

To detach the cells, the growth medium was removed, and the wells were washed with an EDTA solution (PanEco, Moscow, Russia). Accutase (STEMCELL Technologies, USA) was then added, and the cells were incubated for 5 min and then removed via gentle suspension, centrifuged, and washed once in PBS. The cell suspension was incubated with FITC-labeled anti-CD44, APC-labeled anti-EpCam (Miltenyi Biotech, Macquarie Park, NSW, Australia), FAM maleimide (FAM)-labeled or Cyanine5 maleimide (Cy5)-labeled anti-HER2 and anti-GD2 antibodies (generated as previously described [24]) for 1 h. After incubation, the cells were washed twice in PBS. Measurements were performed using a BD FACS AriaIII flow cytometer–sorter (BD Biosciences, Franklin Lakes, NJ, USA), recording 10^4^ events per sample. The relative fluorescence intensity (RFI) was calculated as the ratio of the fluorescence from cells stained with fluorescently labeled antibodies to the autofluorescence of control unstained cells. The results were processed using the FlowJo software, version 10.8.1.

## 3. Results

### 3.1. Clinical Cohort and RNA Sequencing

**Figure 1:**
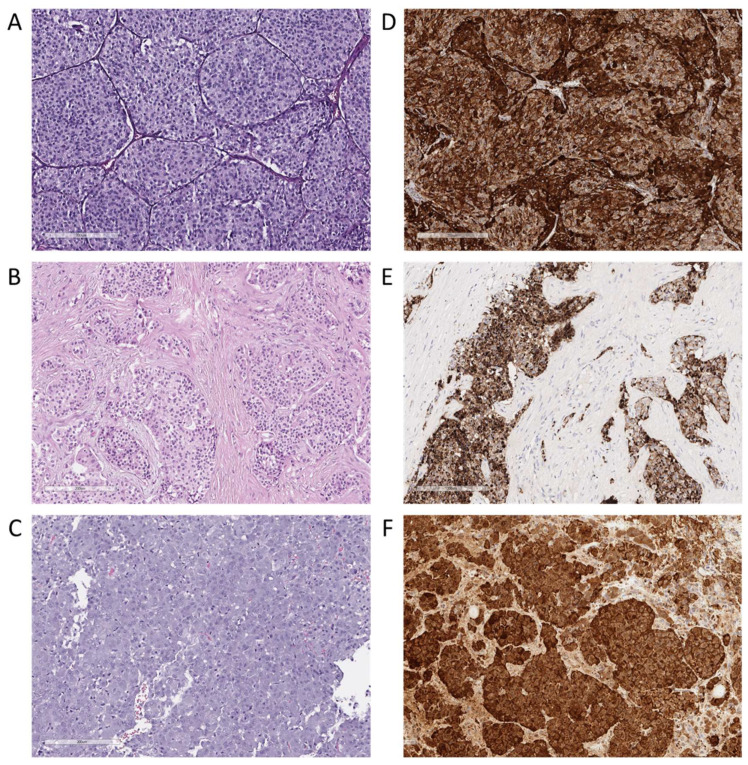
Histological and immunohistochemical analysis of tumor samples: (**A**) lung ACTH-producing NET, (**B**) pancreatic ACTH-producing NET, and (**C**) pituitary ACTH-producing NET, all stained with hematoxylin and eosin, shown at 100× magnification. Immunohistochemical staining results for ACTH are displayed for (**D**) lung NET at 100×, (**E**) pancreatic NET at 100×, and (**F**) pituitary NET at 200× magnification. The scale bar length is 200 µm.

Thirty-six patients (28 females and eight males), aged between 14 and 67 years, with clinical, laboratory, radiological, and histopathological diagnoses of ACTH-dependent hypercortisolism (Cushing’s disease (*n* = 29) or ectopic ACTH-syndrome (*n* = 7)) were included in this study. A summary of the clinical and pathological features of the cohort is shown in Table 1, and representative immunohistochemistry microphotographs are presented in Figure 1.

The pituitary and ectopic tumors in this study showed statistically significant differences in several physiological effects, as detailed in Table 1: morning and late-night plasma ACTH level was about two times higher in the ectopic group; late-night serum and salivary cortisol level was approximately 1.5-fold higher in the ectopic group; and 24-hour urinary free cortisol level was about four times higher in the ectopic group.

RNA isolation and sequencing were performed on the biosamples described above, resulting in twenty-two high-quality RNA sequencing (RNA-seq) profiles for the pituitary ACTH-secreting tumors and seven for the ectopic ACTH-secreting tumors. Additionally, RNA sequencing was carried out on seven specimens of pathologically normal pituitaries, yielding high-quality RNA expression profiles. The experimental RNA-seq data obtained in this study were deposited in the Gene Expression Omnibus (GEO) repository under the accession ID GSE275374.

### 3.2. Grouping of Biosamples by RNA Expression Data

The group of tumor biosamples under analysis was co-investigated alongside seven pathologically normal pituitary specimens profiled in this study, as well as eight healthy lung and eight healthy pancreas RNA-seq profiles obtained from the ANTE database [18]. The latter two groups of the normal samples were selected because the RNA-seq profiles from the ANTE database were previously profiled by us using the same protocols, equipment, and reagents as in the current set of experiments. Furthermore, these ANTE profiles were derived from normal tissues taken from healthy individuals killed in road accidents, making them optimal gene expression normalization controls [25].

**Figure 2:**
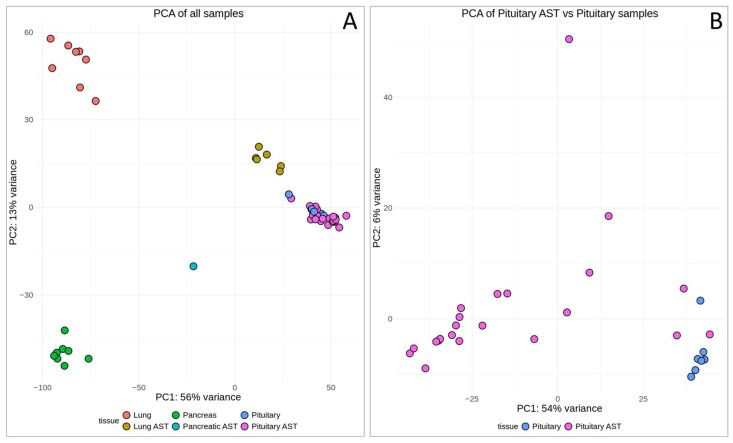
Principal component analysis (PCA) plot of ACTH-secreting tumors and healthy tissue transcriptomic profiles under analysis. (**A**) Comparison of all samples under analysis. (**B**) Comparison of pituitary ACTH-secreting tumors and healthy pituitary biosamples. Each dot represents an individual sample, color-coded by tissue type.

The principal component analysis (PCA) method was used to discern the variance in gene expression across tissue types and pathological states under analysis. The PCA plot (Figure 2A) illustrates the primary axes of variance captured by the first two principal components, PC1 and PC2, which account for 56% and 13% of the variance, respectively. We observed clear-cut separate clustering of the healthy lung and pancreas tissue biosamples and of both types of ectopic ACTH-secreting tumors (lung and pancreatic NETs). Conversely, pituitary ACTH-secreting tumors formed a cluster that partially overlapped with the normal pituitary tissue biosamples. Interestingly, although they were separately positioned, the clusters derived from pituitary and lung ACTH-secreting tumors were located relatively close to each other, while a pancreatic ACTH-secreting tumor sample showed a markedly distinct localization on the plot compared to any other group under analysis.

We then assessed in more detail the relationship between the pituitary control samples and the pituitary ACTH-secreting tumor samples (Figure 2B). On a separate PCA plot, where PC1 and PC2 accounted for 54% and 6% of the variance, respectively, we observed a clearly distinct localization pattern between the pituitary ACTH-secreting tumors and the control samples (Figure 2B). Interestingly, the pituitary ACTH-secreting tumor samples were relatively widely dispersed along the first component (PC1) axis, suggesting a high degree of variability within this group, potentially reflecting different subtypes or stages of the disease. In contrast, the pituitary controls were tightly clustered, indicating a more uniform gene expression pattern within the group of healthy tissues (Figure 2B). Note that six pituitary ACTH-secreting tumor samples were closely clustered with healthy pituitary samples, and these were excluded from further analysis.

### 3.3. Differential Gene Expression Analysis

We then compared gene expression profiles to identify differentially expressed genes (DEGs). First, we identified DEGs specific to (i) pituitary ACTH-secreting tumors vs. normal pituitaries, (ii) lung ACTH-secreting tumors vs. normal lungs, and (iii) a pancreatic ACTH-secreting tumor sample vs. normal pancreatic tissues. Second, we aimed to identify genes specific to ectopic ACTH-secreting tumors. The DEGs found in the respective comparisons were further used to identify statistically and significantly enriched gene ontology (GO) terms and differentially activated intracellular molecular pathways.

**Figure 3:**
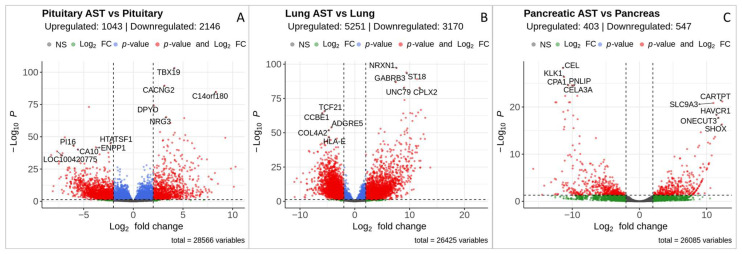
Differentially expressed genes identified for the comparisons of ACTH-secreting tumors with their respective normal tissues. The comparisons of pituitary ACTH-secreting tumors and normal pituitaries (**A**), lung ACTH-secreting tumors and normal lungs (**B**), and a pancreatic ACTH-secreting neuroendocrine tumor and normal pancreas samples (**C**) are shown. The x-axis represents the log_2_ (fold change) of gene expression levels, while the y-axis shows the negative logarithm of the adjusted *p*-value. Red dots indicate genes that meet the *p*-value and fold-change thresholds, as represented by the horizontal and vertical dashed lines. The thresholds are set at an absolute value of log_2_ (fold change) > 2 and a Benjamani–Hochberg FDR-adjusted *p*-value < 0.05.

For the comparison (i) between pituitary ACTH-secreting tumors and pituitary control samples, we identified a total of 3189 DEGs (1043 upregulated and 2146 downregulated genes in pituitary ACTH-secreting tumors), as shown in Figure 3A and Supplementary Table S2. Interestingly, the predominance of downregulated genes may reflect a loss of major molecular functions normally executed by healthy pituitaries.

The gene ontology (GO) enrichment analysis revealed that, compared to healthy pituitary tissues, pituitary ACTH-secreting tumors showed an upregulation of genes involved in ion transport and neuronal activity, suggesting changes in cellular signaling and excitability. In contrast, downregulated genes were associated with developmental processes, growth regulation, and extracellular matrix components, indicating their suppression. Additionally, upregulated genes were linked to membrane channel functions, while downregulated genes were related to receptor activities, pointing to a potential reduction in cellular responsiveness to external signals (Supplementary Figure S1).

For the comparison (ii) between lung ACTH-secreting tumors and normal lung tissue, a total of 8421 DEGs were found, with 5251 up- and 3170 downregulated genes in lung ACTH-secreting tumors (Figure 3B and Supplementary Table S3). Lung ACTH-secreting tumors are neuroendocrine tumors, and this type of cells represents only a minor proportion of healthy lung tissue. Thus, the apparently big difference in gene expression patterns, in addition to tumor-specific alterations, may also represent variability between cell compositions of the lung ACTH-secreting tumors and the overall lung tissue.

The GO analysis showed that lung ACTH-secreting tumors, compared to normal lungs, had upregulated genes involved in neurotransmitter and ion transport processes, indicating enhanced synaptic-like activity and altered ion homeostasis, potentially affecting cellular excitability and signaling. Downregulated genes were linked to immune responses and structural pathways, suggesting reduced extracellular matrix components and local immune activity. In terms of cellular components, upregulated genes were associated with synaptic and vesicular structures, reflecting enhanced neuroendocrine communication. Conversely, downregulated genes were enriched for extracellular matrix elements, indicating decreased structural integrity. For molecular functions, upregulated genes were related to ion channel activities, while downregulated genes involved binding to actin, integrins, and cytokines, pointing to reduced interactions with structural proteins and signaling molecules, potentially affecting adhesion, migration, and communication (Supplementary Figure S2).

Finally, in the comparison (iii) between pancreatic neuroendocrine tumor samples and normal pancreatic tissues, we detected a total of 950 DEGs, including 403 upregulated and 547 downregulated in the pancreatic ACTH-secreting tumor (Figure 3C, Supplementary Table S4). In the latter case, the analysis was based on a single sample, and the results should be considered with caution. The DEG analysis provides an initial overview of the pancreatic ACTH-secreting tumor-specific molecular alterations; however, these results need to be validated in a larger cohort of patients.

The GO analysis revealed that the pancreatic ACTH-secreting tumor exhibited upregulated genes enriched for neuronal and synaptic processes, such as axonogenesis, synaptic plasticity regulation, and neurotransmitter secretion, indicating enhanced neuronal-like activity. In contrast, downregulated genes were linked to metal ion homeostasis and detoxification, including zinc and copper ion regulation. In the “Cellular Components” category, upregulated genes were associated with synaptic and transporter structures, such as the synaptic membrane and ion channel complexes, while downregulated genes were related to extracellular matrix elements and secretory pathways, suggesting reduced matrix integrity and altered secretory functions. For molecular functions, upregulated genes highlighted increased ion transport activities, whereas downregulated genes were involved in peptidase activity and cadherin binding, reflecting functional differences between neuroendocrine tumor cells and normal pancreatic tissue (Supplementary Figure S3).

### 3.4. Common Differential Gene Expression Pattern of ACTH-Secreting Tumors

**Figure 4:**
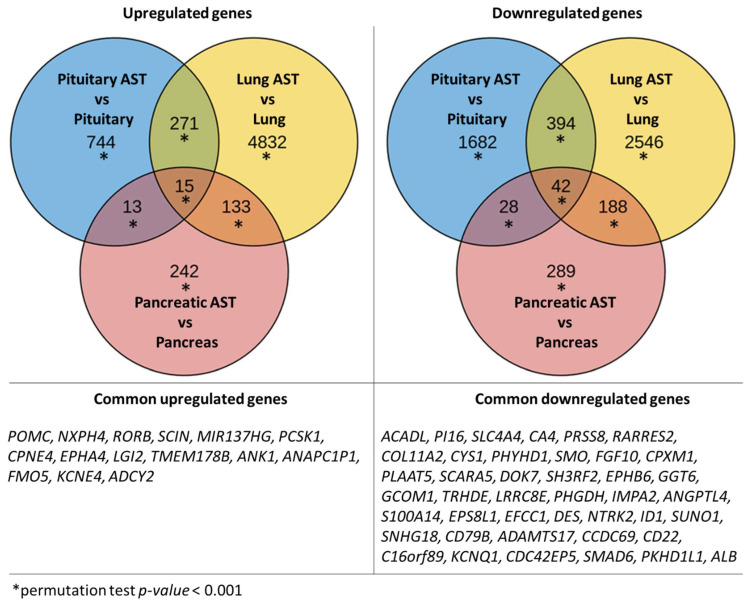
Intersection of differentially regulated genes in pituitary, lung, and pancreatic ACTH-secreting tumors compared to their respective normal tissue controls.

We then compared all DEGs identified in the pituitary, lung and pancreatic ACTH-secreting tumors (Figure 4) and found 15 upregulated and 42 downregulated genes common across all three types of ACTH-secreting tumors (Supplementary Table S5). This intersection of differentially regulated genes across all three ACTH-secreting tumor types was non-random for both upregulated and downregulated genes, as indicated by a permutation test with a *p*-value < 0.001 (Figure 4).

### 3.5. Common Gene Ontology Term Characteristic for ACTH-Secreting Tumors

We found that some GO terms were common for all types of ACTH-secreting tumors under analysis in their comparison with the respective normal tissues (Supplementary Figure S4). Across all ACTH-secreting tumors, gene expression shifts consistently point toward enhanced neuroendocrine and synaptic-like features, as evidenced by increased ion transport, membrane potential regulation, and neurotransmitter receptor activities, alongside a reduction in developmental and structural integrity processes, particularly diminished collagen-containing extracellular matrix components. Notably, this downregulation of building extracellular matrix may be one of the major fundamental features of cancer tissues [25].

### 3.6. Pathway Activation Patterns in ACTH-Secreting Tumors

The pathway activation level (PAL) is an effective quantitative measure of the degree of activation of a specific molecular pathway. It considers both the quantitative expression level of a gene and its functional role in the pathway (e.g., activating, inhibitory, and neutral). Positive PAL values indicate the upregulation of the corresponding molecular processes and vice versa. PAL values can be calculated using both transcriptomic and proteomic data [20]. In this study, we assessed PAL values calculated for ACTH-secreting tumors versus control healthy tissue samples using current experimental RNA-seq profiles.

The analysis revealed distinct but interconnected patterns of pathway activation and inhibition across pituitary, lung, and pancreatic ACTH-secreting tumors. In pituitary ACTH-secreting tumors (Supplementary Figure S5 and Supplementary Table S6), pathways linked to aflatoxin and botulinum toxin detoxification were highly upregulated, reflecting enhanced metabolic and detoxification capabilities. The fibronectin matrix formation pathway also showed strong activation, in line with studies indicating fibronectin as a key molecular marker in pituitary adenomas for over three decades [26]. Additionally, multiple branches of fibroblast growth factor (FGF) signaling were elevated (e.g., the negative regulation of apoptosis and the MAPKKK cascade), suggesting increased migratory potential, survival, and potential interactions with the tumor stroma [27,28]. Conversely, pathways crucial for glycoprotein hormone production and neuronal guidance (e.g., Slit-Robo and Hedgehog) were downregulated, indicating disrupted endocrine and developmental signals.

In lung ACTH-secreting tumors (Supplementary Figure S6 and Supplementary Table S7), enhanced toxin-related (e.g., the botulinum toxin) and opioid signaling pathways coexisted with the suppression of pathways governing cell growth inhibition (e.g., SMAD2/3) and angiogenesis (e.g., VEGFR3). Such changes likely foster tumor proliferation and reduce normal vascular development, potentially contributing to a more aggressive phenotype.

For the pancreatic ACTH-secreting tumor (Supplementary Figure S7, Supplementary Table S8), there was a pronounced activation of neuronal function pathways (e.g., NEUROG3-regulated gene expression in endocrine progenitor cells) and serotonin/melatonin biosynthesis. The upregulation of the FOXA1 transcription factor network and the downregulation of the MTA3 pathway point to significant transcriptional reprogramming, consistent with reports that the MTA3 protein is often upregulated in cancers [29]. Meanwhile, impaired serine biosynthesis, reduced immune signaling (e.g., the FC epsilon receptor), and diminished integrin-mediated adhesion (e.g., α6β1/α6β4 hemidesmosome assembly) indicate a loss of normal pancreatic functionality.

Collectively, these pathway modulations underline the neuroendocrine nature of ACTH-secreting tumors, showcasing their heightened metabolic adaptability, altered survival and proliferative signals, and diminished endocrine homeostasis.

### 3.7. Commonly Regulated Pathways in ACTH-Secreting Tumors

**Figure 5:**
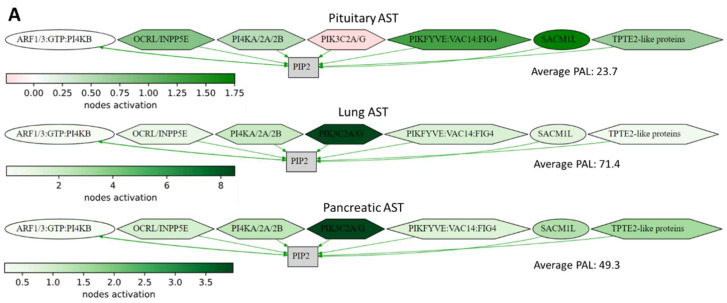

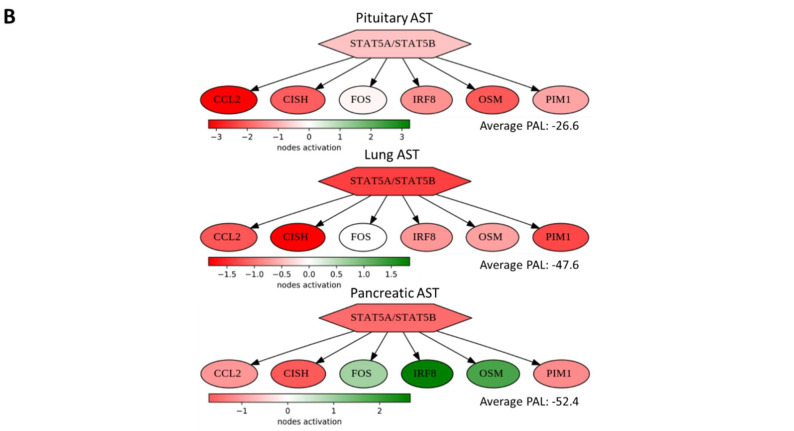
Activation profiles of selected common differentially regulated molecular pathways in the ACTH-secreting tumors for (**A**) the Reactome synthesis of PIPs at the Golgi membrane main pathway and (**B**) the GMCSF-mediated signaling events main pathway. The color reflects the logarithm of the case-to-normal ratio (CNR) of the pathway nodes; the color scale is given (green—upregulated, red—downregulated, and white—intact). Arrows show molecular interactions within a pathway: green stands for activation and red for inhibition. PAL values were calculated for the averaged biosamples in each group.

Some pathways showed a common regulation trend in all types of ACTH-secreting tumors under study compared to the normal tissues (Supplementary Table S9), including 14 upregulated and 13 downregulated pathways. The detailed pathway activation charts of the representative upregulated and downregulated pathways in ACTH-secreting tumors are shown in Figure 5.

### 3.8. Regulation of Expression of the Pro-Opiomelanocortin (POMC) Gene

The overexpression of pro-opiomelanocortin (*POMC*) and the subsequent hypersecretion of adrenocorticotropic hormone (ACTH) are hallmark features of ACTH-secreting tumors, leading to the clinical manifestations of Cushing’s syndrome. The regulation of pro-opiomelanocortin (*POMC*) gene expression involves a complex interplay of transcription factors (TFs) and enhancers that respond to developmental cues and hormonal signals within corticotroph cells of the pituitary gland. *POMC* gene regulation is tightly controlled by specific enhancer sequences located both proximally and distally relative to the gene’s transcription start site. These enhancers harbor binding sites for multiple TFs, including Tpit (also known as Tbx19, T-box transcription factor 19, *TBX19*), Pitx1 (pituitary homeobox 1, *PITX1*), and NeuroD1 (neuronal differentiation 1, *NEUROD1*), which are critical for corticotroph-specific expression [13,30,31].

Pitx1 and Tpit play a fundamental role in this regulation by binding to a composite regulatory element within the *POMC* enhancer, where their cooperative interaction is essential for the transcriptional activation of the gene. Furthermore, the transcription of *POMC* is subject to regulation by hormonal inputs such as corticotropin-releasing hormone (CRH), which activates *POMC* expression through the orphan nuclear receptors of the Nur family—Nurr77 (nuclear receptor subfamily 4 group A member 1, *NR4A1,* also known as Nur77) and NOR1 (nuclear receptor subfamily 4 Group A member 3, *NR4A3*) [32,33].

**Figure 6:**
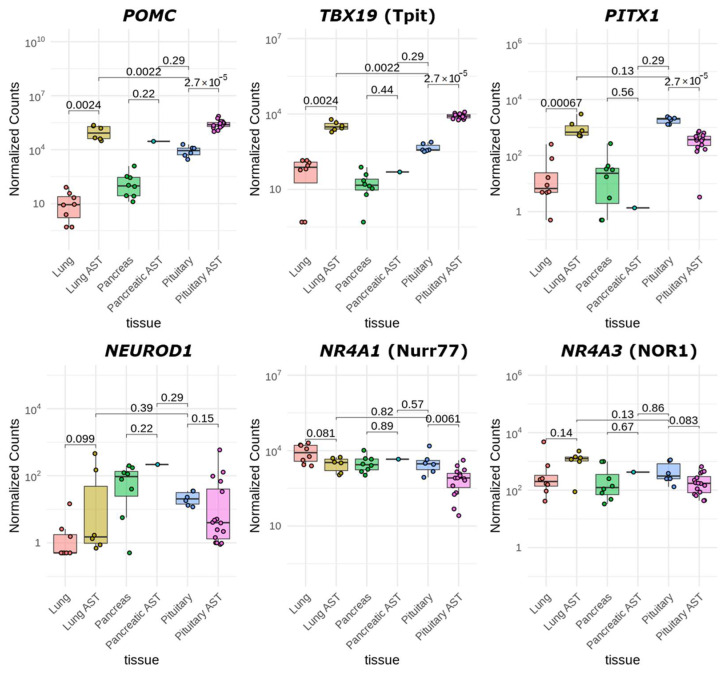
Expression levels of genes regulating *POMC* transcription. Normalized expression levels of the *POMC*, *TBX19*, *PITX1*, *NEUROD1*, *NR4A1*, and *NR4A3* genes in experimental ACTH-secreting tumors and normal tissue samples are shown. Each point represents an individual sample, with the y-axis showing normalized counts on a log10 scale. Boxplots show the median and interquartile ranges for each group. Statistical significance of differences in gene expression levels between groups is indicated by brackets and the respective *p*-values (Wilcoxon rank-sum test).

In this study, the *POMC* gene was markedly overexpressed across all ACTH-secreting tumor samples (Figure 6), exhibiting the highest expression in pituitary ACTH-secreting tumors, where it was elevated ~30-fold relative to normal pituitary tissue. Expression in lung ACTH-secreting tumors was ~12-fold higher, while in pancreatic ACTH-secreting tumor, it was ~3-fold higher than in the pituitary (Figure 6).

Similarly, *TBX19* was significantly overexpressed in pituitary ACTH-secreting tumors (~18-fold higher than in pituitary) and lung ACTH-secreting tumors (~46-fold higher than in normal lung tissue and ~7.5-fold higher than in pituitary); however, it was not overexpressed in our pancreatic ACTH-secreting tumor sample. These findings are consistent with the only previously published data on *TBX19* expression in lung and pancreatic ACTH-secreting tumors, as confirmed via reverse transcription PCR and immunohistochemistry [15].

Interestingly, *PITX1* expression was elevated in lung ACTH-secreting tumors compared to normal lung tissue (~24-fold), yet in both lung and pituitary ACTH-secreting tumors, expression levels were lower than in normal pituitary tissue (~1.7-fold lower in lung and ~5-fold lower in pituitary ACTH-secreting tumors). No *PITX1* expression was detected in our pancreatic ACTH-secreting tumor sample. The expression of the *NEUROD1* transcription factor gene was generally low across all samples, with notable heterogeneity observed in lung and pituitary ACTH-secreting tumor samples. In each of these tumors, two distinct groups were observed, characterized by relatively high and low expression levels of this gene. The expression of Nur family receptors (Nurr77 and NOR1) was uniformly high across all samples, with no significant variations in NOR1, whereas Nurr77 was expressed at a ~4.4-fold lower level in pituitary ACTH-secreting tumors compared to normal pituitary tissue.

### 3.9. Genes and Molecular Processes Specifically Regulated in Lung ACTH-Secreting Tumors

**Figure 7:**
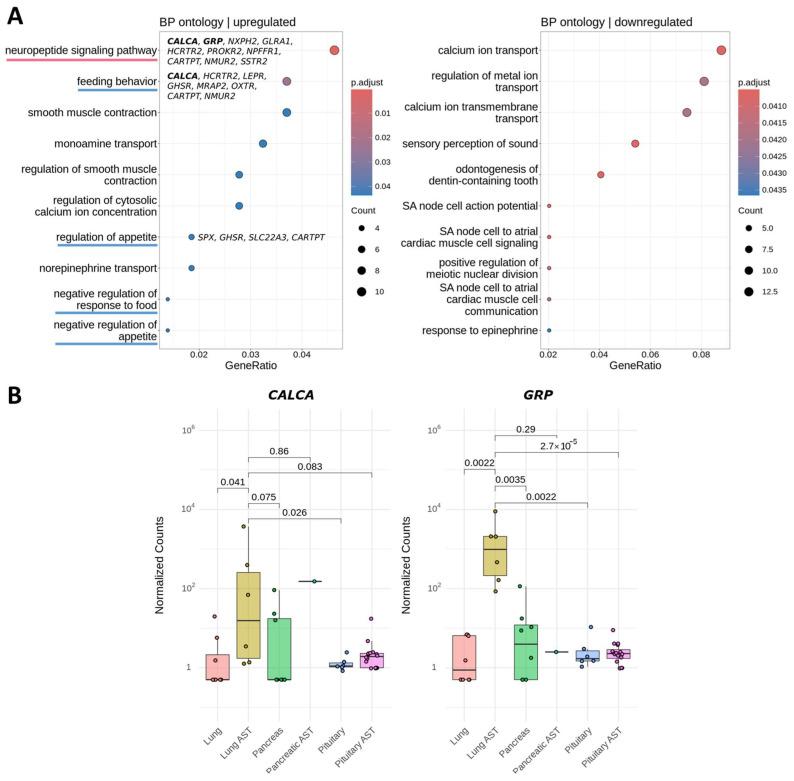
Biological process (BP) ontology analysis of differentially expressed genes (DEGs) identified in lung ACTH-secreting tumors versus pituitary ACTH-secreting tumors, normal lungs, and normal pituitaries (**A**). Upregulated pathways include neuropeptide signaling, feeding behavior, and the regulation of appetite, with specific genes such as *CALCA*, *GRP*, and others highlighted. The GeneRatio on the x-axis represents the proportion of DEGs associated with each GO term. The size of the bubbles indicates the number of genes involved, and the color gradient reflects the adjusted *p*-value, with redder shades indicating more statistically significant enrichment. (**B**) Expression levels of *CALCA* and *GRP* across all analyzed tissues, showing normalized counts on a log_10_ scale. Each point represents an individual sample, while boxplots depict the median and interquartile ranges for each group. Statistical significance of differences in gene expression levels between groups is indicated by brackets and the respective *p*-values (Wilcoxon rank-sum test).

Since lung ACTH-secreting tumors are understudied and their molecular diagnostics are currently unfeasible, we focused on the identification of genes and molecular processes specifically regulated in these tumors. To this end, we identified genes that were differentially regulated in lung ACTH-secreting tumors compared to pituitary ACTH-secreting tumors, normal lungs, and normal pituitaries. In total, we identified 494 upregulated and 202 downregulated genes in lung ACTH-secreting tumors (Supplementary Table S10). These genes were enriched in a number of specific processes related to neuroendocrine cell physiology (Figure 7A). For example, downregulated genes were enriched in calcium ion transport, and upregulated genes were involved in neuropeptide signaling (Figure 7A).

Unexpectedly, we also found that many top GO terms enriched for lung AST-upregulated genes were related to feeding behavior and the regulation of appetite (Figure 7A). Interestingly, some of the related genes encode secretory proteins that can be identified in the bloodstream, such as GRP, the gastrin-releasing peptide (Figure 7A). Its expression was markedly (100-fold or more) increased in the lung ACTH-secreting tumor samples in comparison to all other tumors and normal tissues under analysis (Figure 7B). This may suggest its potential usefulness as the biomarker for detecting lung ACTH-secreting tumors, where regular instrumental detection and visualization is extremely challenging. Furthermore, in both lung and pancreatic ACTH-secreting tumors, we found increased expression of the CALCA gene, which encodes the peptide hormone calcitonin (Figure 7B). This may also suggest its potential applicability for the molecular diagnostics of these pathologies.

### 3.10. Fusion Gene Analysis in ACTH-Secreting Tumors

We then assessed whether the gene expression profiles of ACTH-secreting tumors established here included any fusion transcripts. Fusion transcripts may originate from genomic rearrangements such as translocations, deletions, and duplications; alternatively, they may be formed due to read-through transcription and/or splicing aberrations. Using the STAR-Fusion software and specific filtering settings, we identified a total of 91 candidate fusion transcripts of protein-coding genes with canonical splice site junctions (Supplementary Table S11).

**Figure 8:**
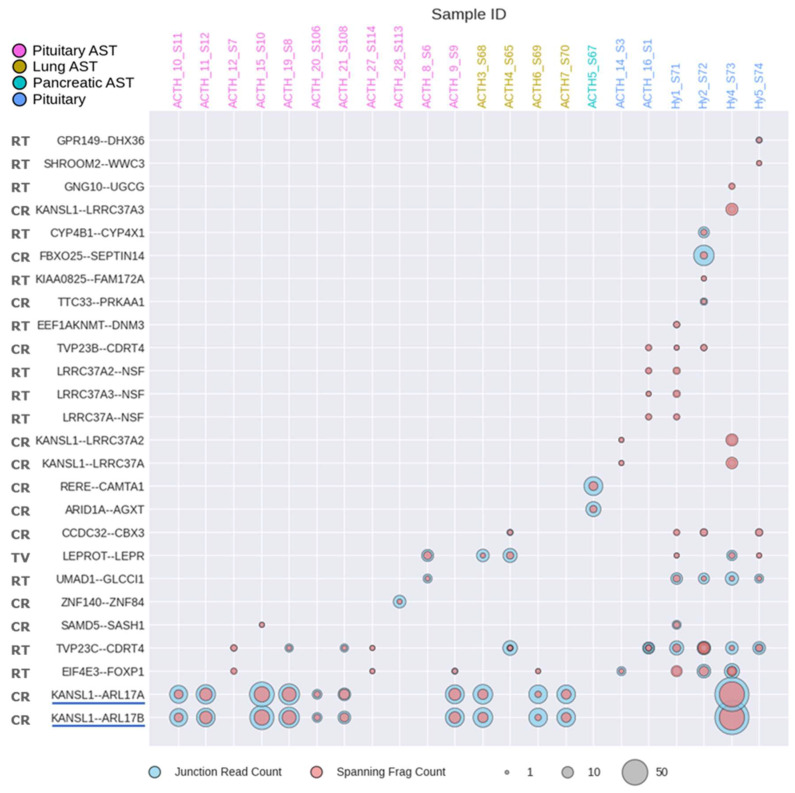
Distribution of gene fusion candidates in the analyzed samples. Each sample is represented on the x-axis, while the y-axis lists the gene fusions identified in each sample. Light blue dots indicate the junction read count (the number of reads containing the fusion junction), and pink dots indicate the spanning fragment count (the number of reads flanking the fusion region) for each fusion in the sample. The size of the dots is proportional to these counts. The following designations were used: RT—read-through transcript, CR—likely chromosomal rearrangement, and TV—likely transcript variant. The most frequent fusion candidates are highlighted.

Of the 91 fusion candidates, 26 were uniquely named and verified against several known oncological databases, including OncoKB, the Mitelman Database, COSMIC, and ChimerDB 4.0. Notably, the *SAMD5*–*SASH1* fusion was detected in almost all databases. Our analysis revealed several fusions described in at least one database, such as *CCDC32*–*CBX3* and *KIAA0825*–*FAM172A*, among others. Furthermore, six new gene fusions, previously unrepresented in these databases, were identified: *GPR149*–*DHX36*, *FBXO25*–*SEPTIN14*, *KANSL1*–*LRRC37A3*, *RERE*–*CAMTA1*, *ARID1A*–*AGXT*, and *ZNF140*–*ZNF84*. Fourteen fusion candidates were detected in more than one biosample (Figure 8). Among them, the group with two candidate fusions, (i) *KANSL1*–*ARL17A* and *KANSL1*–*ARL17B,* most likely represented one real fusion. The same was true for the groups with fusions (ii) *KANSL1*–*LRRC37A2 and KANSL1*–*LRRC37A and* (iii) *LRRC37A*–*NSF, LRRC37A2*–*NSF, and LRRC37A3*–*NSF.* Fusion candidate *LEPROT*–*LEPR* represented *LEPR* gene transcript variants.

Among the 26 uniquely named fusion candidates, 12 included moieties of neighboring genes and, therefore, likely represented read-through transcripts. In turn, 13 fusion candidates represented more distantly located genes, thus supporting the possibility of their emergence due to chromosomal rearrangements (Supplementary Table S11).

### 3.11. Modeling of Targeted Drug Sensitivity of ACTH-Secreting Tumors and Experimental Validation

Using the RNA-seq profiles of ACTH-secreting tumors, we modeled the sensitivity profiles of individual biosamples to 170 cancer-targeted therapeutics. This was performed using the Oncobox method [22], which has proven useful in the clinic for predicting the sensitivity of individual tumors to targeted drugs based on the expression of target genes and the activation of target molecular pathways [20].

The metric termed the balanced efficiency score (BES) reflects the expected efficacy of a drug and can take on both positive and negative values. Positive values here indicate an overall upregulation of drug target genes and pathways in an individual tumor, and vice versa. When comparing different drugs, a higher BES value suggests greater expected efficacy of a drug; conversely, a lower BES value suggests lower expected efficacy of treatment with the respective drug. This makes it possible to create a personalized drug rating for an individual tumor sample.

**Figure 9:**
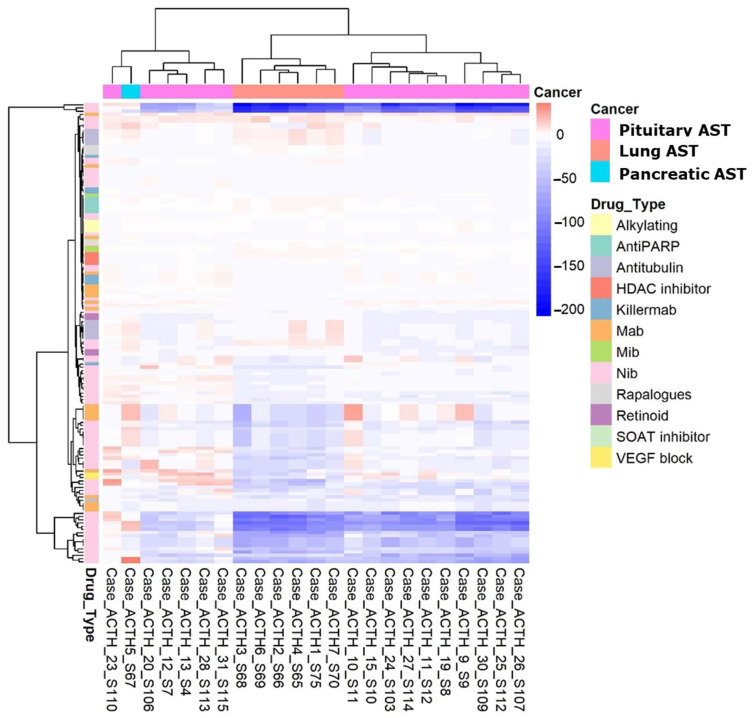
Heatmap for lung, pancreatic, and pituitary ACTH-secreting tumors based on cancer-targeted drug *balanced efficiency score* (BES) values calculated using the Oncobox software (https://app.oncobox.com, accessed on 25 June 2024) for individual gene expression profiles of ACTH-secreting tumor biosamples under analysis.

We used this approach to simulate the efficiency of 170 targeted therapeutics for the treatment of different types of ACTH-secreting tumors (Figure 9; Supplementary Table S12). According to the modeled profiles of drug sensitivity, the ACTH-secreting tumors were divided into two major clusters. Cluster 1, encompassing 30% of the ACTH-secreting tumors, included the pituitary ACTH-secreting tumors and one pancreatic ACTH-secreting tumor sample, while cluster 2, comprising 70%, included the remaining pituitary ACTH-secreting tumors and all lung ACTH-secreting tumors, with the lung ACTH-secreting tumors forming a distinct subcluster (Figure 9). However, several therapeutics showed high predicted efficacy in both clusters in the majority of the samples (Table 2). For example, the RET inhibitors pralsetinib and selpercatinib were among the top ten most effective drugs predicted for both pituitary and lung ACTH-secreting tumors, with *RET* gene upregulation in 15/16 and 6/6 investigated cases, respectively. The IL6R inhibitor tocilizumab was also predicted to be effective in the majority of cases due to upregulation of *IL6R* expression in 15/16 pituitary ACTH-secreting tumors and 6/6 lung ACTH-secreting tumors (Table 2).

**Figure 10:**
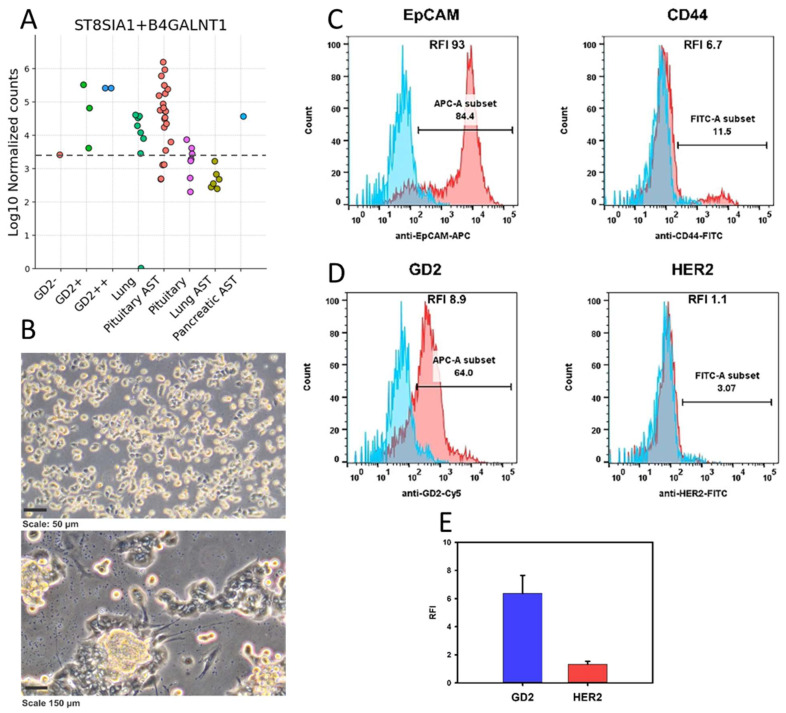
(**A**) Distribution of the 2-gene signature score for GD2 expression based on the transcription of the *ST8SIA1* and *B4GALNT1* genes. (**B**) Phase-contrast microscopy of tumor cells isolated from a biopsy from a patient with an ACTH-secreting pituitary tumor (pAST-3). Cells were photographed using an Axiovert 40 CFL inverted microscope and a Nikon D5000 digital camera. (**C**) Flow cytometry analysis of CD44 and EpCAM expression in cells from the biopsy of patient pAST1 with a pituitary ACTH-secreting tumor. Blue peak—autofluorescence of the unstained cells, red peak—fluorescence of the antibody-stained cells. Marker indicates the fraction of positively stained cells (%). (**D**) Flow cytometry analysis of GD2 and HER2 expression in cells from the biopsy of patient pAST1 with a pituitary ACTH-secreting tumor. Blue peak—autofluorescence of the unstained cells, red peak—fluorescence of the antibody-stained cells. Marker indicates the fraction of positively stained cells (%). (**E**) Relative fluorescence intensity (RFI) expression of ganglioside GD2 and HER2 by primary cell cultures obtained from biopsies of 3 patients with pituitary ACTH-secreting tumor (patients pAST1, pAST2 and pAST3).

In addition, we simulated the expected efficacy of an emerging class of anticancer drugs that target ganglioside GD2 molecules. This type of therapy is currently approved for clinical use in neuroblastoma [34] but is also being used or considered as an experimental therapy for triple-negative breast cancer [35], medulloblastoma [36], and glioblastoma [37]. Since the immunohistochemical detection of GD2 molecules in FFPE tumor biosamples is challenging, we recently developed a 2-gene signature that provides information about the GD2-positive or GD2-negative status of the biosamples being analyzed [23]. Using this diagnostic 2-gene signature, we found that the majority of pituitary ACTH-secreting tumors showed an enhanced signature score similar to the reference GD2-positive controls, significantly higher than the score calculated for normal pituitary tissue (Figure 10A). Interestingly, this was not the case for the ectopic (lung) ACTH-secreting tumors.

We then experimentally tested this hypothesis. To this end, we obtained primary cell cultures from fresh surgical biosamples of ACTH-secreting tumors from three new unrelated patients diagnosed with pituitary macroadenomas at the Endocrinology Research Center, Moscow. Since the literature suggests that pituitary ACTH-secreting tumors may contain both EpCAM-positive tumor cells [38] and CD44-positive tumor stromal fibroblast-like cells [39,40], we quantified the populations of EpCAM+ and CD44+ cells via flow cytometry analysis. In addition, the cells were stained with a GD2-specific antibody [24] and also with HER2-specific antibodies as negative controls (all ACTH-secreting tumors were predicted to be HER2-negative using the Oncobox method), Figure 10B–E. As a result, we observed 84–98% of EpCAM-positive cells and 7–13% of CD44-positive cells, suggesting that most of the population belongs to the pituitary adenoma cells. Only 3–9% of the cells were HER2-positive, and 55–64% of the cells were GD2-positive (Figure 10B–E, Supplementary Figure S8). Thus, our results confirmed the bioinformatically predicted GD2-positive status of pituitary adenoma cells and suggest the possibility of using GD2-targeted therapy for the treatment of pituitary ACTH-secreting tumors.

## 4. Discussion

This is the first study aiming to evaluate gene expression profiles across various ACTH-secreting tumors to identify new targets for differential diagnostics and for the treatment of ACTH-dependent hypercortisolism. We revealed biological similarities in these tumors, including 15 genes overexpressed in all three types of ACTH-secreting tumors (*POMC*, *NXPH4*, *RORB*, *SCIN*, *MIR137HG*, *PCSK1*, *CPNE4*, *EPHA4*, *LGI2*, *TMEM178B*, *ANK1*, *ANAPC1P1*, *FMO5*, *KCNE4*, and *ADCY2*), as well as differentially expressed mRNAs in lung ACTH-secreting tumor encoding secretory proteins (GRP (gastrin releasing peptide) and CALCA (calcitonin)) that may potentially be detected in circulation. We identified six new gene fusions previously unrepresented in specific databases and found targets for existing medications in other cancer types that may be tested in patients with ACTH-secreting tumors.

Our data partly supports the neuroendocrine origin of pituitary ACTH-secreting tumors [41] showing similar upregulated neuropeptide signaling pathways. The commonly overexpressed genes—*NXPH4* (neurexophilin 4), *RORB* (RAR related orphan receptor B), lncRNA, EPHA4 (EPH receptor A4)—exhibit the common neuroendocrine features of ACTH-producing tumors as well [42,43,44,45,46].

However, it has become increasingly evident over recent decades that pituitary ACTH-secreting tumors differ considerably among themselves in terms of secretory parameters and responses to medical therapy [47]. Our data supported their heterogeneity as shown in the principal component analysis; even though we investigated microadenomas, these tissue samples did not include aggressive macroadenomas.

The overexpression of pro-opiomelanocortin (POMC) and the subsequent hypersecretion of adrenocorticotropic hormone (ACTH) are hallmark features of ACTH-secreting tumors, leading to the clinical manifestations of CS, increased morbidity, and mortality [3,5,11]. The *POMC* gene was markedly overexpressed across all ACTH-secreting tumor samples. Our analysis also revealed the overexpression of human prohormone convertase 1 (*PCSK1*) which cleaves POMC to produce ACTH and melanocortin [48] in all ACTH-secreting tumors. The commonly overexpressed genes that may be involved in tumor secretion include *SCIN* (scinderin) and *CPNE4* (calcium-dependent phospholipid-binding protein). Usually, secretory vesicles are prevented from entering release sites on the plasma membrane due to the presence of a cortical actin filament network. An increase in intracellular calcium activates scinderin, consequently resulting in the severing of actin filament and the local dissociation of actin filament networks. This allows the movement of secretory vesicles to release sites on the plasma membrane [49]. *CPNE4* may play a role in calcium-mediated intracellular processes [50].

Interestingly, PITX1 transcription factor gene expression was elevated in lung ACTH-secreting tumors compared to normal lung tissue, yet in both lung and pituitary ACTH-secreting tumors, expression levels were lower than in normal pituitary tissue. No *PITX1* expression was detected in our pancreatic ACTH-secreting tumor sample. The expression of the transcription factor gene *NEUROD1* was generally low across all samples, with notable heterogeneity observed in lung and pituitary ACTH-secreting tumors. In each of these tumors, two distinct groups were observed, characterized by relatively high and low expression levels of this gene. The expression of Nur family receptors (Nurr77 and NOR1) was uniformly high across all samples, with no significant variations, except for Nur77, which was expressed at ~4.4-fold lower levels in pituitary ACTH-secreting tumors compared to normal pituitary tissue.

In pituitary ACTH-secreting tumors, the remarkable overexpression of *TBX19* (Tpit) suggests its pivotal role in driving *POMC* transcription. TBX19 is known to interact with enhancer elements within the *POMC* gene and is essential for the corticotroph-specific expression of *POMC.* The reduced expression of PITX1 and Nur77 in these tumors indicates that while these factors can contribute to *POMC* regulation, their decreased levels do not impede the overexpression of *POMC*, possibly due to the compensatory overactivity of TBX19. In pulmonary ACTH-secreting tumors, both *TBX19* and *PITX1* are overexpressed relative to normal lung tissue, suggesting a cooperative role in enhancing *POMC* transcription. The high level of NOR1 further supports the involvement of hormone-responsive pathways in *POMC* overexpression. However, this scenario was not effective in our pancreatic ACTH-secreting tumor sample, where the absence of *TBX19* and *PITX1* overexpression points to alternative mechanisms driving the expression of *POMC*. Pitx1 and Tpit play a fundamental role in this regulation by binding to a composite regulatory element within the *POMC* enhancer, where their cooperative interaction is essential for the transcriptional activation of the gene. Furthermore, the transcription of *POMC* is subject to regulation by hormonal inputs such as corticotropin-releasing hormone (CRH), which activates *POMC* expression through the orphan nuclear receptors of the Nur family—Nurr77 (nuclear receptor subfamily 4 group A member 1, *NR4A1*, also known as Nur77) and NOR1 (nuclear receptor subfamily 4 group A member 3, *NR4A3*) [32,33].

In this study, we report gene fusions in ACTH-secreting tumors, with *KANSL1*–*ARL17A* emerging as the most frequently observed. This fusion corresponds to a common haplotype involving a locally rearranged genomic region predominantly identified in European populations [51]. Both *KANSL1* and *ARL17A* are located on the reverse strand of chromosome 17q21.31. *KANSL1* encodes an evolutionarily conserved nuclear protein, which is a subunit of the MLL1 and NSL1 complexes involved in histone H4 acetylation and p53 Lys120 acetylation [52]. The *ARL17A* gene encodes a member of the ADP-ribosylation factor family, which regulates membrane trafficking and vesicular transport [53]. An earlier study also identified *KANSL1*–*ARL17A* in various tumor samples and suggested that it may represent a cancer predisposition germline fusion specific to Europeans [54].

Interestingly, several of the gene fusions identified in our study were also detected in normal pituitary gland samples, including the recurrent *KANSL1*–*ARL17A* fusion. The presence of these rearrangements in healthy tissue highlights the need for caution when interpreting gene fusions solely as potential oncogenic drivers. While it is possible that such events represent benign or passenger alterations, larger-scale studies and functional assays are warranted to determine whether any of these fusions have physiological relevance or predispose cells to future neoplastic transformation.

To identify potential new biomarkers for differential diagnostics, we looked for known secretory peptides used as biomarkers in neuroendocrine tumors [9]. We found the overexpression of the gastrin-releasing peptide in lung ACTH-secreting tumor NET samples compared to all other tumors and normal tissues under analysis. In both lung and pancreatic ACTH-secreting tumors, we found an increased expression of the calcitonin gene *CALCA*, which may also suggest its potential applicability for the molecular diagnostics of these pathologies. Whether these proteins could be useful biomarkers that can detected in peripheral circulation requires further testing.

There is an increasing demand for more precise therapies and relevant biological markers that correlate with treatment responses in patients with EAS and CD. The current approach includes somatostatin analogs, mTOR inhibitors, chemotherapy, and peptide receptor radionuclide therapy, which have significantly enhanced management in cases of advanced disease progression [9]. There exists a recognized clinical need for novel personalized therapeutic strategies. Using RNA-seq profiles of ACTH-secreting tumors, we modeled the sensitivity profiles of individual samples to cancer-targeted therapeutics. We predicted RET inhibitors pralsetinib and selpercatinib among the top ten most effective in both pituitary and lung ACTH-secreting tumors, with the RET gene upregulated in 15/16 pituitary and 6/6 lung ACTH-secreting tumor cases investigated. The IL6R inhibitor tocilizumab was also predicted to be effective in the majority of cases due to the upregulation of *IL6R* expression in 15/16 pituitary and 6/6 lung ACTH-secreting tumors. Interestingly, IL6R signaling through the JAK2/STAT3/MMP9 axis was previously reported to promote the invasion of pituitary adenoma cells [55]. Thus, our results suggest studying these targeted cancer drugs for the treatment of advanced cases of ACTH-secreting tumors when an effective alternative to surgery may be considered for testing.

Due to the apparent molecular heterogeneity present in individual tumors, it is imperative to characterize the biological features of as many individual ACTH-secreting tumors samples as possible at both the genomic and transcriptomic levels, thereby enhancing our understanding of the disease’s mechanistic background and aiding the development of new therapeutic solutions. A significant portion of the genes differentially expressed in our study were either poorly characterized or had unknown functions. In addition, we simulated the expected efficacy of an emerging class of cancer drugs targeted against ganglioside GD2 molecules. This type of therapy is currently approved for clinical use in neuroblastoma [34] and as an experimental therapy for triple-negative breast cancer [35], for medulloblastoma [36], and for glioblastoma [37]. Using our diagnostic 2-gene signature [23], we found that most pituitary ACTH-secreting tumors showed an enhanced signature score similar to the reference GD2-positive controls, significantly exceeding the score calculated for normal pituitary tissues. This GD2-positive status of pituitary ACTH-secreting tumors was further confirmed experimentally using primary cell cultures isolated from unrelated fresh pituitary ACTH-secreting tumor tissues. However, lung or pancreatic ACTH-secreting NETs did not show promise for this intervention.

## 5. Conclusions

We performed a transcriptome analysis of twenty-two pituitary, one pancreatic, and six lung ACTH-secreting tumors, compared to seven healthy pituitary, eight pancreatic, and eight lung tissue samples, respectively. The results are publicly accessible with GEO repository accession ID GSE120795. In all ACTH-secreting tumors, with the exception of the pancreatic sample, we found an overexpression of the TBX19 and PITX1 transcription factor genes, which promote the overexpression of POMC, the precursor gene for ACTH production. However, the transcriptional profiles of ectopic lung ACTH-secreting tumors were significantly different, showing marked overexpression of the gastrin-releasing peptide and calcitonin genes, which may be potentially useful for diagnostic purposes. We identified several transcriptionally activated molecular pathways in ACTH-secreting tumors that might be targetable with existing medications for cancer treatment. Additionally, we predicted and experimentally validated that corticotropinomas express ganglioside GD2 molecules, offering new therapeutic targets.

## Figures and Tables

**Table 1:** Summary of clinical and pathological features of patients included in the study cohort.

Characteristics	Cushing’s Disease	Ectopic ACTH Syndrome	*p*-Value
Total	29	7	−
Male/Female	5/24	3/4	0.117
Age at the time of diagnosis (years)	44 [26; 50]	32 [24; 42]	0.379
Disease duration prior to initial diagnosis (months)	16 [3; 62]	17 [9; 24]	0.984
Body mass index	32.5 [26.8; 35.3]	30 [25.9; 33.2]	0.289
Presence of arterial hypertension	24 (82.8%)	6 (85.7%)	0.848
Presence of diabetes mellitus	8 (27.6%)	3 (42.9%)	0.650
Presence of osteoporosis and fragility fractures	6 (20.7%)	3 (42.9%)	0.333
Morning plasma ACTH	46.3 [37.5; 76.3]	104 [100.3; 140.5]	<0.001
Late-night plasma ACTH	49.4 [33.2; 67.7]	104.4 [78.3; 174.9]	<0.001
Late-night serum cortisol	717 [538.6; 934.6]	1144 [885; 1339]	0.004
Late-night salivary cortisol	29 [15.5; 52.9]	67.2 [45; 120.1]	0.009
24 h urinary free cortisol	1479.6 [871.4; 2264.6]	5542 [2144; 7000]	0.001
With/without visualization of tumor on MRI or CT in the first imaging	17/12	2/5	−
Remission after surgery	25 (86.2%)	6 (85.7%)	−
Relapse after surgery	2 (6.9%)	1 (14.3%)	−

**Table 2:** Top ten positions of cancer-targeted drug/drug classes with the highest predicted balanced efficiency score (BES) for lung, pancreatic, and pituitary ACTH-secreting tumors and the proportion of patients with upregulated molecular targets.

	Drug	Mean BES	Proportion of Patients with Upregulated Molecular Target (s)
Pituitary AST	Tocilizumab	6.66	*IL6R* (15/16)
	Aflibercept	5.56	*PGF* (12/16), *VEGFA* (11/16), *VEGFB* (9/16)
	Bevacizumab	2.38	*VEGFA* (11/16)
	Pralsetinib/Selpercatinib	2.29	*RET* (15/16)
	Claudiximab	1.76	*CLDN18* (15/16)
	Ganetespib	0.83	*HSP90AA1* (16/16)
	Ibritumomab tiuxetan/Obinutuzumab/Ofatumumab/Rituximab	0.6	*MS4A1* (10/16)
	Ixazomib	0.42	*PSMB1* (13/16), *PSMB2* (15/16), *PSMB5* (14/16)
	Carfilzomib	0.36	*PSMB1* (13/16), *PSMB10* (3/16), *PSMB2* (15/16), *PSMB5* (14/16), *PSMB8* (12/16), *PSMB9* (6/16)
	Bortezomib	0.25	*PSMB1* (13/16), *PSMB5* (14/16)
Lung AST	Pralsetinib/Selpercatinib	5.61	*RET* (6/6)
	Tocilizumab	5.14	*IL6R* (6/6)
	Cabazitaxel/Eribulin/Ixabepilone	4.53	*TUBB1* (5/6), *TUBB2A* (6/6), *TUBB2B* (6/6), *TUBB3* (5/6), *TUBB4A* (3/6), *TUBB4B* (1/6)
	Ceritinib/Alectinib	3.91	*ALK* (6/6)
	Docetaxel/Paclitaxel	3.49	*TUBB1* (5/6), *TUBB2A* (6/6), *TUBB2B* (6/6), *TUBB4A* (3/6), *TUBB4B* (1/6)
	Vinblastine/Vincristine/ Vindesine	1.91	*TUBA1A* (5/6), *TUBA1B* (5/6), *TUBA4A* (1/6), *TUBA4B* (2/6), *TUBA8* (6/6), *TUBB1* (5/6), *TUBB3* (5/6), *TUBB4A* (3/6)
	Romidepsin	1.48	*HDAC1* (6/6), *HDAC2* (6/6), *HDAC3* (6/6)
	Panobinostat	1.33	*HDAC1* (6/6), *HDAC2* (6/6), *HDAC3* (6/6), *HDAC5* (4/6), *HDAC6* (6/6)
	PARP inhibitors	1.11	*PARP1* (6/6), *PARP2* (6/6), *PARP3* (5/6), *PARP4* (2/6)
	Everolimus	0.92	*FKBP1A* (1/6), *MTOR* (6/6)
Pancreatic AST	Imatinib	35.36	*ABL2*, *BCR*, *KIT*, *PDGFRA*, *PDGFRB*, *RET*
	Masitinib	34.22	*KIT*, *LYN*, *PDGFRA*, *PDGFRB*
	Sunitinib	18.97	*CSF1R*, *KIT*, *PDGFRA*, *PDGFRB*
	Anti-EGFR mAbs	16.71	*EGFR*
	Pazopanib	15.77	*KIT*, *PDGFRA*, *PDGFRB*
	Tivozanib	15.19	*KIT*, *PDGFRA*, *PDGFRB*
	Brigatinib	12.19	*ALK*, *EGFR*
	Adagrasib/Sotorasib	11.54	*KRAS*
	Anti-EGFR small molecule inhibitors	8.47	*EGFR*
	Trebananib	7.11	*ANGPT1*, *ANGPT2*

## Data Availability

Primary AST patient RNA sequencing data were deposited in the Gene Expression Omnibus (GEO) repository under the accession ID GSE275374.

## Supplementary Materials

The following supporting information can be downloaded at https://www.mdpi.com/article/10.3390/cancers17040658/s1, Figure S1: Gene ontology enrichment analysis for “Biological Processes” (**A**), “Cellular Components”, (**B**) and “Molecular Functions”; (**C**) categories for differentially expressed genes (DEGs) of pituitary ASTs; Figure S2: Gene ontology enrichment analysis for “Biological Processes” (**A**), “Cellular Components”, (**B**) and “Molecular Functions”; (**C**) categories for differentially expressed genes (DEGs) of lung ASTs; Figure S3: Gene ontology enrichment analysis for “Biological Processes” (**A**), “Cellular Components”, (**B**) and “Molecular Functions”; (**C**) categories for differentially expressed genes (DEGs) of a pancreatic AST; Figure S4: Comparative gene ontology enrichment analysis for “Biological Processes” (BP), “Cellular Components” (CC), and “Molecular Functions” (MF) for differentially expressed genes (DEGs) in three tumor-control pairs; Figure S5: Top 10 upregulated and downregulated molecular pathways in pituitary ASTs compared to normal pituitary tissue; Figure S6: Top 10 upregulated and downregulated molecular pathways in lung ASTs compared to normal lung tissue; Figure S7: Top 10 upregulated and downregulated molecular pathways in a pancreatic AST compared to normal pancreas tissue; Figure S8: Flow cytometry analysis of CD44, EpCAM, GD2, and HER2 expression in cells from the biopsy of patient with pituitary ASTs; Table S1: Clinical and pathological characteristics of patients included in the study; Table S2: Differential gene transcription analysis results comparing a pituitary AST and pituitary control samples; Table S3: Differential gene transcription analysis results comparing a lung AST and normal lung tissue samples; Table S4: Differential gene transcription analysis results comparing a pancreatic AST sample and normal pancreatic tissues; Table S5: Common differentially expressed genes (DEGs) identified across pituitary, lung, and pancreatic ASTs; Table S6: Pathway activation analysis in pituitary ASTs; Table S7: Pathway activation analysis in lung ASTs; Table S8: Pathway activation analysis in a pancreatic AST; Table S9: Common pathways across all types of ASTs compared to control samples; Table S10: Differentially expressed genes (DEGs) identified in lung ASTs compared to pituitary ASTs, normal lung, and normal pituitary tissues; Table S11: Fusion transcript candidates identified in AST samples; Table S12: Simulation of targeted therapeutic efficiency for different types of ASTs.

## References

[1] Reincke M., Fleseriu M. (2023). Cushing Syndrome: A Review. JAMA.

[2] Pivonello R., Isidori A.M., De Martino M.C., Newell-Price J., Biller B.M.K., Colao A. (2016). Complications of Cushing’s Syndrome: State of the Art. Lancet Diabetes Endocrinol..

[3] Belaya Z.E., Rozhinskaya L.Y., Dragunova N.V., Dzeranova L.K., Marova E.I., Arapova S.D., Molitvoslovova N.N., Zenkova T.S., Melnichenko G.A., Dedov I.I. (2013). Metabolic Complications of Endogenous Cushing: Patient Selection for Screening. Obe. Metab..

[4] Feelders R., Sharma S., Nieman L. (2015). Cushing’s Syndrome: Epidemiology and Developments in Disease Management. Clin. Epidemiol..

[5] Toivanen S., Leijon H., Arola A., Soinio M., Hämäläinen P.O., Metso S., Knutar O., Koivikko M., Ebeling T., Moilanen L. (2021). Characteristics and Outcomes of the Finnish Ectopic ACTH Syndrome Cohort. Endocrine.

[6] Grober Y., Grober H., Wintermark M., Jane J.A., Oldfield E.H. (2018). Comparison of MRI Techniques for Detecting Microadenomas in Cushing’s Disease. J. Neurosurg..

[7] Freda P.U., Beckers A.M., Katznelson L., Molitch M.E., Montori V.M., Post K.D., Vance M.L. (2011). Pituitary Incidentaloma: An Endocrine Society Clinical Practice Guideline. J. Clin. Endocrinol. Metab..

[8] Fukuoka H., Shichi H., Yamamoto M., Takahashi Y. (2020). The Mechanisms Underlying Autonomous Adrenocorticotropic Hormone Secretion in Cushing’s Disease. Int. J. Mol. Sci..

[9] Young J., Haissaguerre M., Viera-Pinto O., Chabre O., Baudin E., Tabarin A. (2020). MANAGEMENT OF ENDOCRINE DISEASE: Cushing’s Syndrome Due to Ectopic ACTH Secretion: An Expert Operational Opinion. Eur. J. Endocrinol..

[10] Davi’ M.V., Cosaro E., Piacentini S., Reimondo G., Albiger N., Arnaldi G., Faggiano A., Mantovani G., Fazio N., Piovesan A. (2017). Prognostic Factors in Ectopic Cushing’s Syndrome Due to Neuroendocrine Tumors: A Multicenter Study. Eur. J. Endocrinol..

[11] Golounina O.O., Belaya Z.E., Rozhinskaya L.Y., Pikunov M.Y., Markovich A.A., Dzeranova L.K., Marova E.I., Kuznetsov N.S., Fadeev V.V., Melnichenko G.A. (2022). Survival Predictors in Patients with Ectopic Acth Syndrome. Probl. Endokrinol. (Mosk)..

[12] Quentien M.H., Barlier A., Franc J.L., Pellegrini I., Brue T., Enjalbert A. (2006). Pituitary Transcription Factors: From Congenital Deficiencies to Gene Therapy. J. Neuroendocrinol..

[13] Lamolet B., Pulichino A.M., Lamonerie T., Gauthier Y., Brue T., Enjalbert A., Drouin J. (2001). A Pituitary Cell-Restricted T Box Factor, Tpit, Activates POMC Transcription in Cooperation with Pitx Homeoproteins. Cell.

[14] Drouin J. (2022). The Corticotroph Cells from Early Development to Tumorigenesis. J. Neuroendocrinol..

[15] Uccella S., Leoni E., Kaiser S., Maragliano R., Valerio A., Libera L., Tanda M.L., Volante M., Diviani D., La Rosa S. (2023). Heterogeneity of TPIT Expression in ACTH-Secreting Extra-Pituitary Neuroendocrine Tumors (NETs) Supports the Existence of Different Cellular Programs in Pancreatic and Pulmonary NETs. Virchows Arch..

[16] Ergin S., Kherad N., Alagoz M. (2022). RNA Sequencing and Its Applications in Cancer and Rare Diseases. Mol. Biol. Rep..

[17] Toader C., Dobrin N., Tataru C.-I., Covache-Busuioc R.-A., Bratu B.-G., Glavan L.A., Costin H.P., Corlatescu A.D., Dumitrascu D.-I., Ciurea A.V. (2023). From Genes to Therapy: Pituitary Adenomas in the Era of Precision Medicine. Biomedicines.

[18] Suntsova M., Gaifullin N., Allina D., Reshetun A., Li X., Mendeleeva L., Surin V., Sergeeva A., Spirin P., Prassolov V. (2019). Atlas of RNA Sequencing Profiles for Normal Human Tissues. Sci. Data.

[19] Haas B.J., Dobin A., Li B., Stransky N., Pochet N., Regev A. (2019). Accuracy Assessment of Fusion Transcript Detection via Read-Mapping and de Novo Fusion Transcript Assembly-Based Methods. Genome Biol..

[20] Buzdin A., Tkachev V., Zolotovskaia M., Garazha A., Moshkovskii S., Borisov N., Gaifullin N., Sorokin M., Suntsova M. (2021). Using Proteomic and Transcriptomic Data to Assess Activation of Intracellular Molecular Pathways. Adv. Protein Chem. Struct. Biol..

[21] Zolotovskaia M.A., Tkachev V.S., Guryanova A.A., Simonov A.M., Raevskiy M.M., Efimov V.V., Wang Y., Sekacheva M.I., Garazha A.V., Borisov N.M. (2022). OncoboxPD: Human 51 672 Molecular Pathways Database with Tools for Activity Calculating and Visualization. Comput. Struct. Biotechnol. J..

[22] Tkachev V., Sorokin M., Garazha A., Borisov N., Buzdin A. (2020). Oncobox Method for Scoring Efficiencies of Anticancer Drugs Based on Gene Expression Data. Methods Mol. Biol..

[23] Sorokin M., Kholodenko I., Kalinovsky D., Shamanskaya T., Doronin I., Konovalov D., Mironov A., Kuzmin D., Nikitin D., Deyev S. (2020). RNA Sequencing-Based Identification of Ganglioside GD2-Positive Cancer Phenotype. Biomedicines.

[24] Kalinovsky D.V., Kibardin A.V., Kholodenko I.V., Svirshchevskaya E.V., Doronin I.I., Konovalova M.V., Grechikhina M.V., Rozov F.N., Larin S.S., Deyev S.M. (2022). Therapeutic Efficacy of Antibody-Drug Conjugates Targeting GD2-Positive Tumors. J. Immunother. Cancer.

[25] Sorokin M., Buzdin A.A., Guryanova A., Efimov V., Suntsova M.V., Zolotovskaia M.A., Koroleva E.V., Sekacheva M.I., Tkachev V.S., Garazha A. (2023). Large-Scale Assessment of Pros and Cons of Autopsy-Derived or Tumor-Matched Tissues as the Norms for Gene Expression Analysis in Cancers. Comput. Struct. Biotechnol. J..

[26] Ozyurt E., Sönmez H., Süer S., Kökoğlu E. (1996). The Prognostic Importance of Fibronectin and Sialic Acid Levels in Human Pituitary Adenomas. Cancer Lett..

[27] Marques P., Barry S., Carlsen E., Collier D., Ronaldson A., Grieve J., Dorward N., Mendoza N., Nair R., Muquit S. (2021). The Expression of Neural Cell Adhesion Molecule and the Microenvironment of Pituitary Neuroendocrine Tumours. J. Neuroendocrinol..

[28] Marques P., Barry S., Carlsen E., Collier D., Ronaldson A., Awad S., Dorward N., Grieve J., Mendoza N., Muquit S. (2019). Pituitary Tumour Fibroblast-Derived Cytokines Influence Tumour Aggressiveness. Endocr. Relat. Cancer.

[29] Brüning A., Blankenstein T., Jückstock J., Mylonas I. (2014). Function and Regulation of MTA1 and MTA3 in Malignancies of the Female Reproductive System. Cancer Metastasis Rev..

[30] Lamolet B., Poulin G., Chu K., Guillemot F., Tsai M.J., Drouin J. (2004). Tpit-Independent Function of NeuroD1(BETA2) in Pituitary Corticotroph Differentiation. Mol. Endocrinol..

[31] Poulin G., Lebel M., Chamberland M., Paradis F.W., Drouin J. (2000). Specific Protein-Protein Interaction between Basic Helix-Loop-Helix Transcription Factors and Homeoproteins of the Pitx Family. Mol. Cell. Biol..

[32] Philips A., Lesage S., Gingras R., Maira M.-H., Gauthier Y., Hugo P., Drouin J. (1997). Novel Dimeric Nur77 Signaling Mechanism in Endocrine and Lymphoid Cells. Mol. Cell. Biol..

[33] Martens C., Bilodeau S., Maira M., Gauthier Y., Drouin J. (2005). Protein-Protein Interactions and Transcriptional Antagonism between the Subfamily of NGFI-B/Nur77 Orphan Nuclear Receptors and Glucocorticoid Receptor. Mol. Endocrinol..

[34] Persaud N.V., Park J.A., Cheung N.K. (2024). V High-Risk Neuroblastoma Challenges and Opportunities for Antibody-Based Cellular Immunotherapy. J. Clin. Med..

[35] Shao C., Anand V., Andreeff M., Battula V.L. (2022). Ganglioside GD2: A Novel Therapeutic Target in Triple-Negative Breast Cancer. Ann. N. Y. Acad. Sci..

[36] Ciccone R., Quintarelli C., Camera A., Pezzella M., Caruso S., Manni S., Ottaviani A., Guercio M., Del Bufalo F., Quadraccia M.C. (2024). GD2-Targeting CAR T-Cell Therapy for Patients with GD2+ Medulloblastoma. Clin. Cancer Res..

[37] Golinelli G., Grisendi G., Prapa M., Bestagno M., Spano C., Rossignoli F., Bambi F., Sardi I., Cellini M., Horwitz E.M. (2020). Targeting GD2-Positive Glioblastoma by Chimeric Antigen Receptor Empowered Mesenchymal Progenitors. Cancer Gene Ther..

[38] Kodani Y., Kawata M., Suga H., Kasai T., Ozone C., Sakakibara M., Kuwahara A., Taga S., Arima H., Kameyama T. (2022). EpCAM Is a Surface Marker for Enriching Anterior Pituitary Cells From Human Hypothalamic-Pituitary Organoids. Front. Endocrinol. (Lausanne).

[39] Kinugasa Y., Matsui T., Takakura N. (2014). CD44 Expressed on Cancer-Associated Fibroblasts Is a Functional Molecule Supporting the Stemness and Drug Resistance of Malignant Cancer Cells in the Tumor Microenvironment. Stem Cells.

[40] Musielak M., Piwocka O., Kulcenty K., Ampuła K., Adamczyk B., Piotrowski I., Fundowicz M., Kruszyna-Mochalska M., Suchorska W.M., Malicki J. (2022). Biological Heterogeneity of Primary Cancer-Associated Fibroblasts Determines the Breast Cancer Microenvironment. Am. J. Cancer Res..

[41] Cassarino M.F., Ambrogio A.G., Cassarino A., Terreni M.R., Gentilini D., Sesta A., Cavagnini F., Losa M., Pecori Giraldi F. (2018). Gene Expression Profiling in Human Corticotroph Tumours Reveals Distinct, Neuroendocrine Profiles. J. Neuroendocrinol..

[42] Born G., Breuer D., Wang S., Rohlmann A., Coulon P., Vakili P., Reissner C., Kiefer F., Heine M., Pape H.-C. (2014). Modulation of Synaptic Function through the α-Neurexin–Specific Ligand Neurexophilin-1. Proc. Natl. Acad. Sci. USA.

[43] Yang Z., Wei B., Qiao A., Yang P., Chen W., Zhen D., Qiu X. (2022). A Novel EZH2/NXPH4/CDKN2A Axis Is Involved in Regulating the Proliferation and Migration of Non-Small Cell Lung Cancer Cells. Biosci. Biotechnol. Biochem..

[44] Cook D.N., Kang H.S., Jetten A.M. (2015). Retinoic Acid-Related Orphan Receptors (RORs): Regulatory Functions in Immunity, Development, Circadian Rhythm, and Metabolism. Nucl. Recept. Res..

[45] Shen H., Wang L., Ge X., Jiang C.F., Shi Z.M., Li D.M., Liu W.T., Yu X., Shu Y.Q. (2016). MicroRNA-137 Inhibits Tumor Growth and Sensitizes Chemosensitivity to Paclitaxel and Cisplatin in Lung Cancer. Oncotarget.

[46] Guella I., Sequeira A., Rollins B., Morgan L., Torri F., van Erp T.G.M., Myers R.M., Barchas J.D., Schatzberg A.F., Watson S.J. (2013). Analysis of MiR-137 Expression and Rs1625579 in Dorsolateral Prefrontal Cortex. J. Psychiatr. Res..

[47] da Silva-Júnior R.M.P., Bueno A.C., Martins C.S., Coelli-Lacchini F., Ozaki J.G.O., de Almeida-E-Silva D.C., Marrero-Gutiérrez J., Dos Santos A.C., Garcia-Peral C., Machado H.R. (2023). Integrating Methylome and Transcriptome Signatures Expands the Molecular Classification of the Pituitary Tumors. J. Clin. Endocrinol. Metab..

[48] Jansen E., Ayoubi T.A.Y., Meulemans S.M.P., Van de Ven W.J. (1995). Neuroendocrine-Specific Expression of the Human Prohormone Convertase 1 Gene. Hormonal Regulation of Transcription through Distinct CAMP Response Elements. J. Biol. Chem..

[49] Rodríguez Del Castillo A., Vitale M.L., Tchakarov L., Trifaró J.M. (1992). Human Platelets Contain Scinderin, a Ca(2+)-Dependent Actin Filament-Severing Protein. Thromb. Haemost..

[50] Creutz C.E., Tomsig J.L., Snyder S.L., Gautier M.-C., Skouri F., Beisson J., Cohen J. (1998). The Copines, a Novel Class of C2 Domain-Containing, Calciumdependent, Phospholipid-Binding Proteins Conserved from Paramecium to Humans. J. Biol. Chem..

[51] Boettger L.M., Handsaker R.E., Zody M.C., McCarroll S.A. (2012). Structural Haplotypes and Recent Evolution of the Human 17q21.31 Region. Nat. Genet..

[52] Li X., Wu L., Corsa C.A.S., Kunkel S., Dou Y. (2009). Two Mammalian MOF Complexes Regulate Transcription Activation by Distinct Mechanisms. Mol. Cell.

[53] Seixas E., Barros M., Seabra M.C., Barral D.C. (2013). Rab and Arf Proteins in Genetic Diseases. Traffic.

[54] Zhou J.X., Yang X., Ning S., Wang L., Wang K., Zhang Y., Yuan F., Li F., Zhuo D.D., Tang L. (2017). Identification of KANSARL as the First Cancer Predisposition Fusion Gene Specific to the Population of European Ancestry Origin. Oncotarget.

[55] Feng J., Yu S.-Y., Li C.-Z., Li Z.-Y., Zhang Y.-Z. (2016). Integrative Proteomics and Transcriptomics Revealed That Activation of the IL-6R/JAK2/STAT3/MMP9 Signaling Pathway Is Correlated with Invasion of Pituitary Null Cell Adenomas. Mol. Cell. Endocrinol..

