# Supplementary material for: Transcriptome-Wide Analysis of Pituitary and Ectopic Adrenocorticotropic Hormone-Secreting Tumors
Source: Cancers (Basel). 2025 Feb 15;17(4):658. doi: 10.3390/cancers17040658 (PMC11852724; doi:10.3390/cancers17040658)
Supplement: Supplementary file 1 [file cancers-17-00658-s001.zip › Supplementary_figures.pdf]

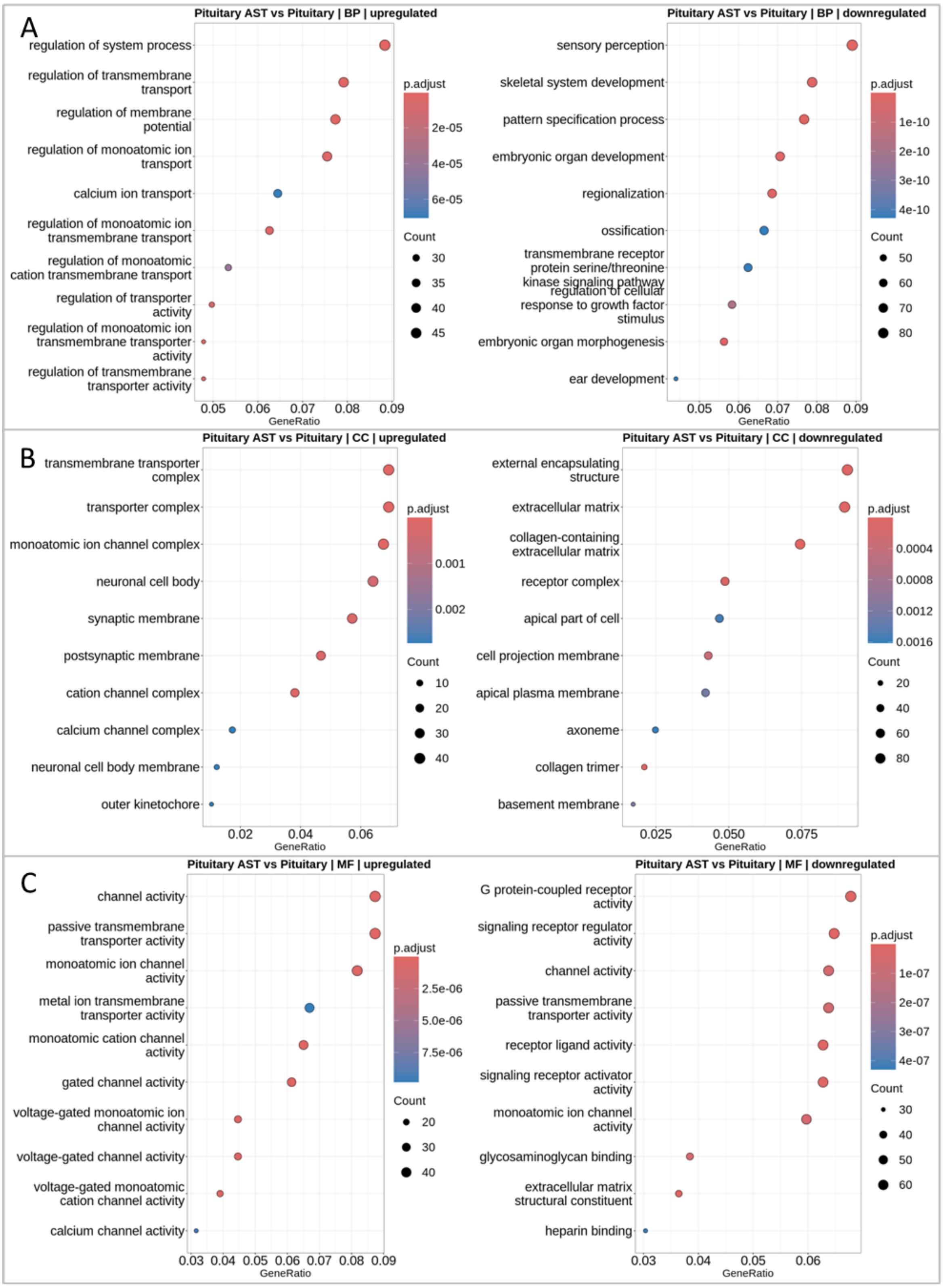

Figure S1. Gene Ontology enrichment analysis for "Biological Processes" (A), "Cellular Components" (B) and "Molecular Functions" (C) categories for differentially expressed genes (DEGs) of pituitary ASTs. The *GeneRatio* on the X-axis represents the proportion of DEGs associated with each GO term. The size of the bubbles indicates the number of genes involved, and the color gradient reflects the adjusted *p-value*, with redder shades indicating more statistically significant enrichment.

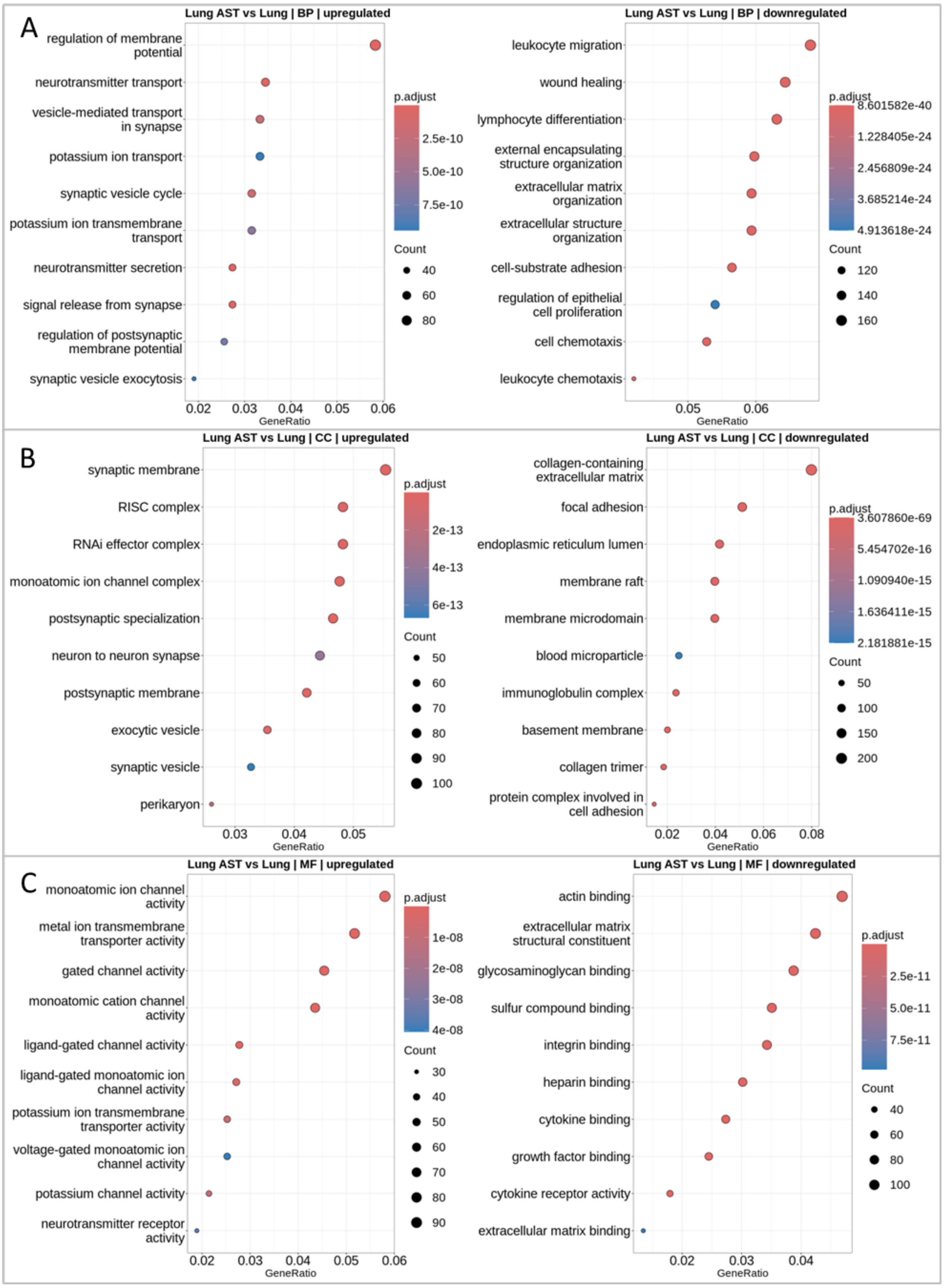

Figure S2. Gene Ontology enrichment analysis for "Biological Processes" (A), “Cellular Components" (B) and "Molecular Functions" (C) categories for differentially expressed genes (DEGs) of lung ASTs. The *GeneRatio* on the X-axis represents the proportion of DEGs associated with each GO term. The size of the bubbles indicates the number of genes involved, and the color gradient reflects the adjusted *p-value*, with redder shades indicating more statistically significant enrichment.

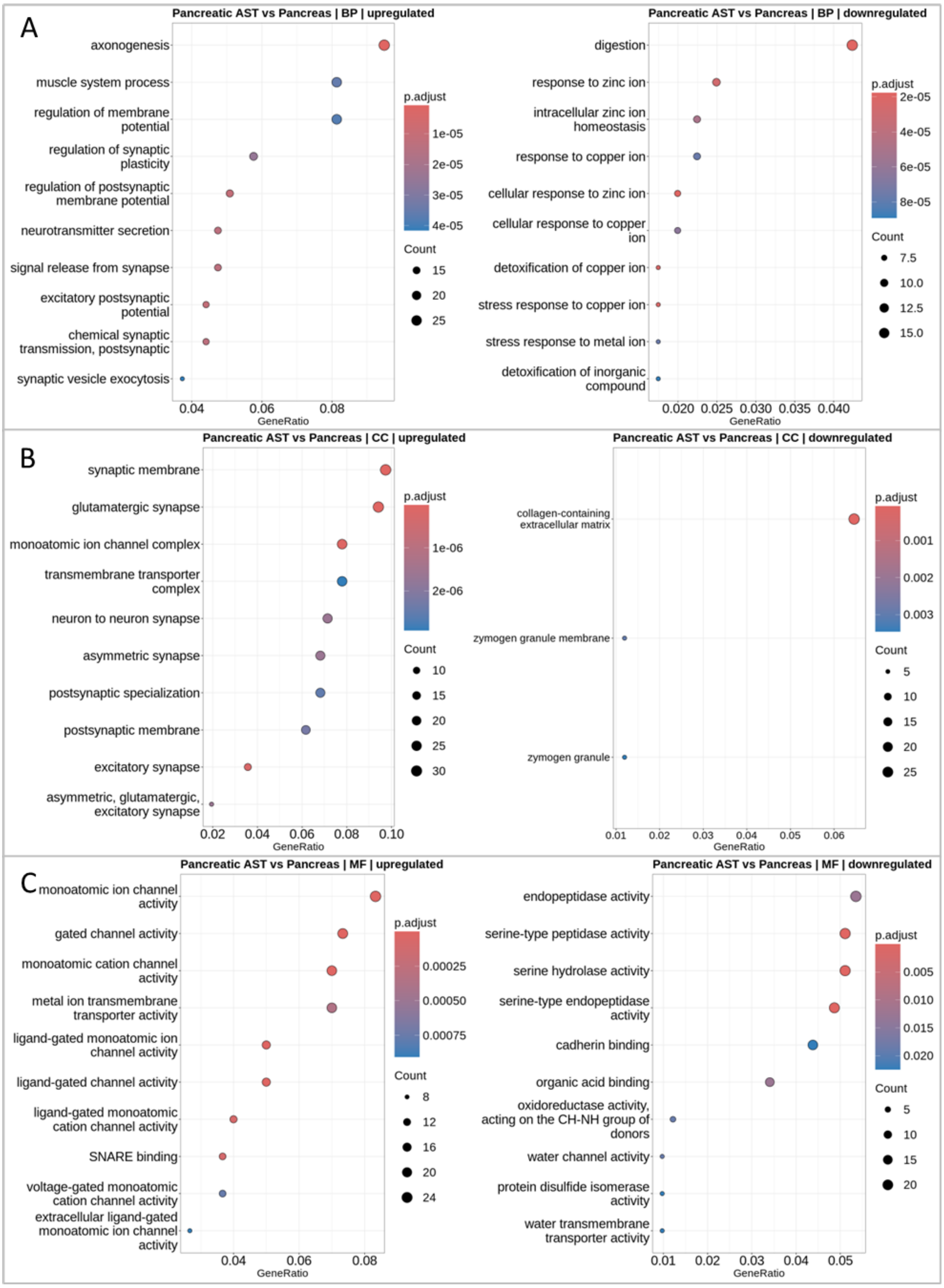

Figure S3. Gene Ontology enrichment analysis for "Biological Processes" (A), “Cellular Components" (B) and "Molecular Functions" (C) categories for differentially expressed genes (DEGs) of pancreatic AST. The *GeneRatio* on the X-axis represents the proportion of DEGs associated with each GO term. The size of the bubbles indicates the number of genes involved, and the color gradient reflects the adjusted *p-value*, with redder shades indicating more statistically significant enrichment.

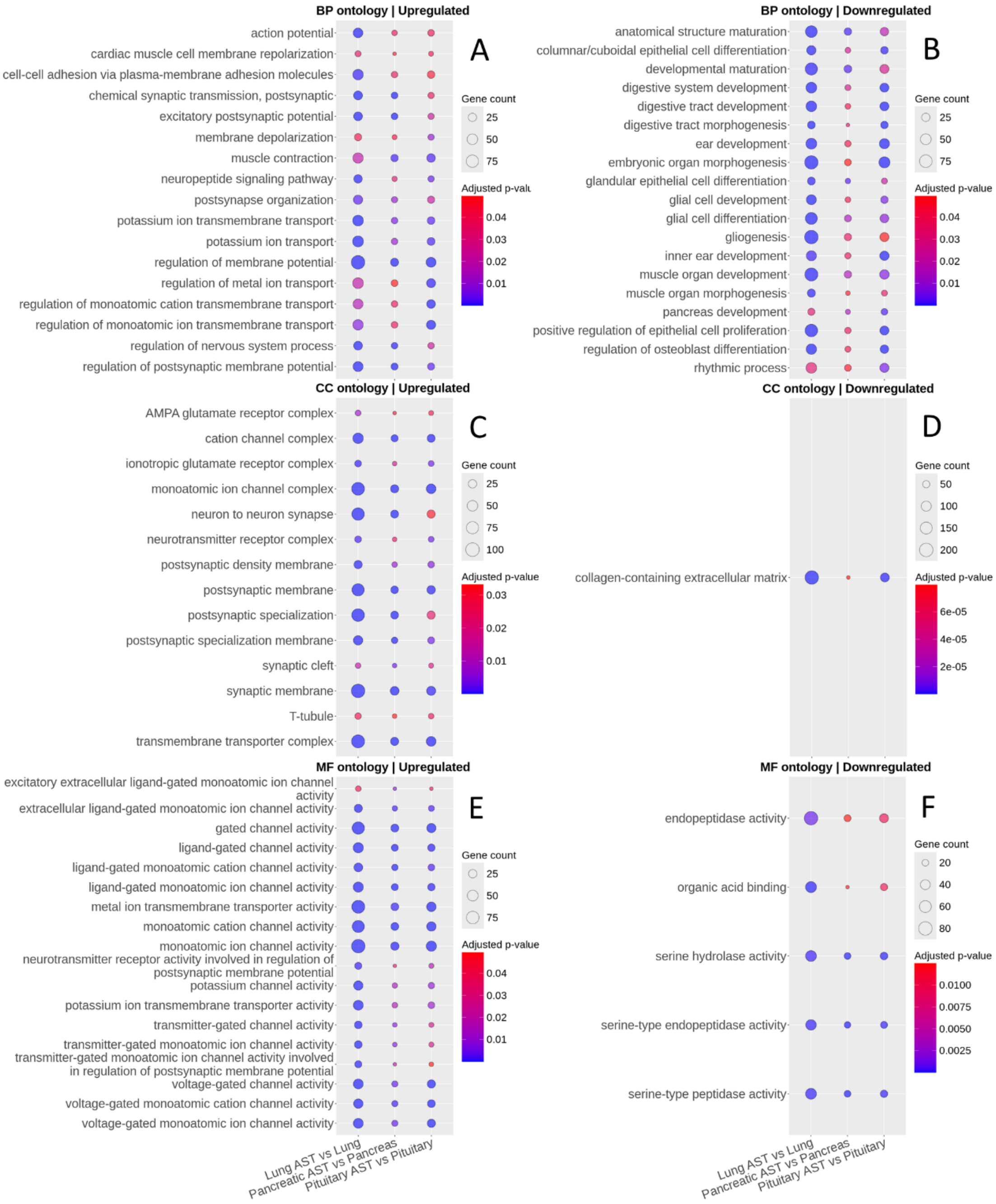

Figure S4. Comparative gene ontology enrichment analysis for "Biological Processes" (BP), "Cellular Components" (CC), and "Molecular Functions" (MF) ontologies for differentially expressed genes (DEGs) in three tumor-control pairs. Panels A, C, and E represent the enriched GO terms for upregulated DEGs, while panels B, D, and F represent those for downregulated DEGs. The size of the circles indicates the gene count, and the color gradient represents the adjusted p-value for significance, with red indicating higher significance.

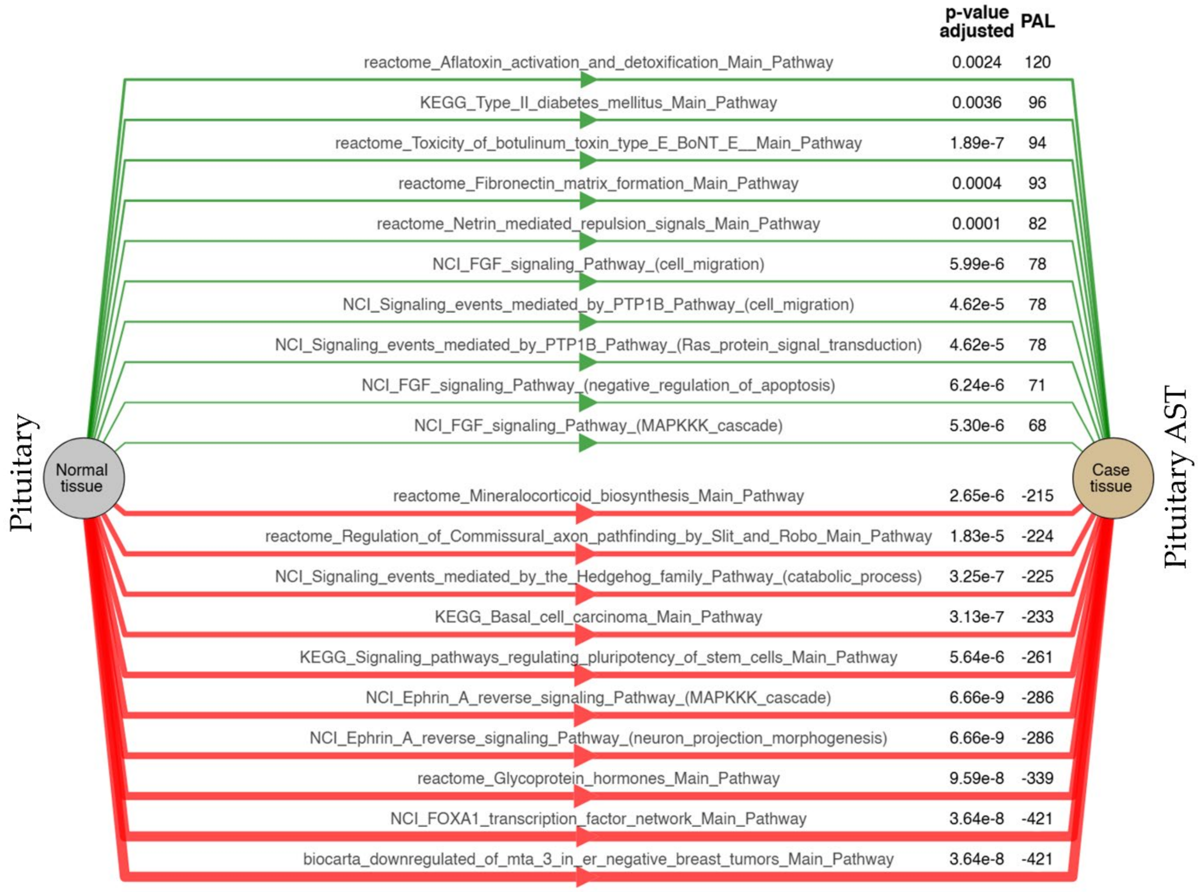

Figure S5. Top 10 upregulated and downregulated molecular pathways in pituitary ASTs compared to normal pituitary tissue. Pathway Activation Level (PAL) values for the top differentially activated molecular pathways are shown. Pathways with increased activation are highlighted in green and have positive PAL values, while inhibited pathways are highlighted in red with negative PAL values. FDR-adjusted p-values indicate statistical significance of the differential regulation of pathways.

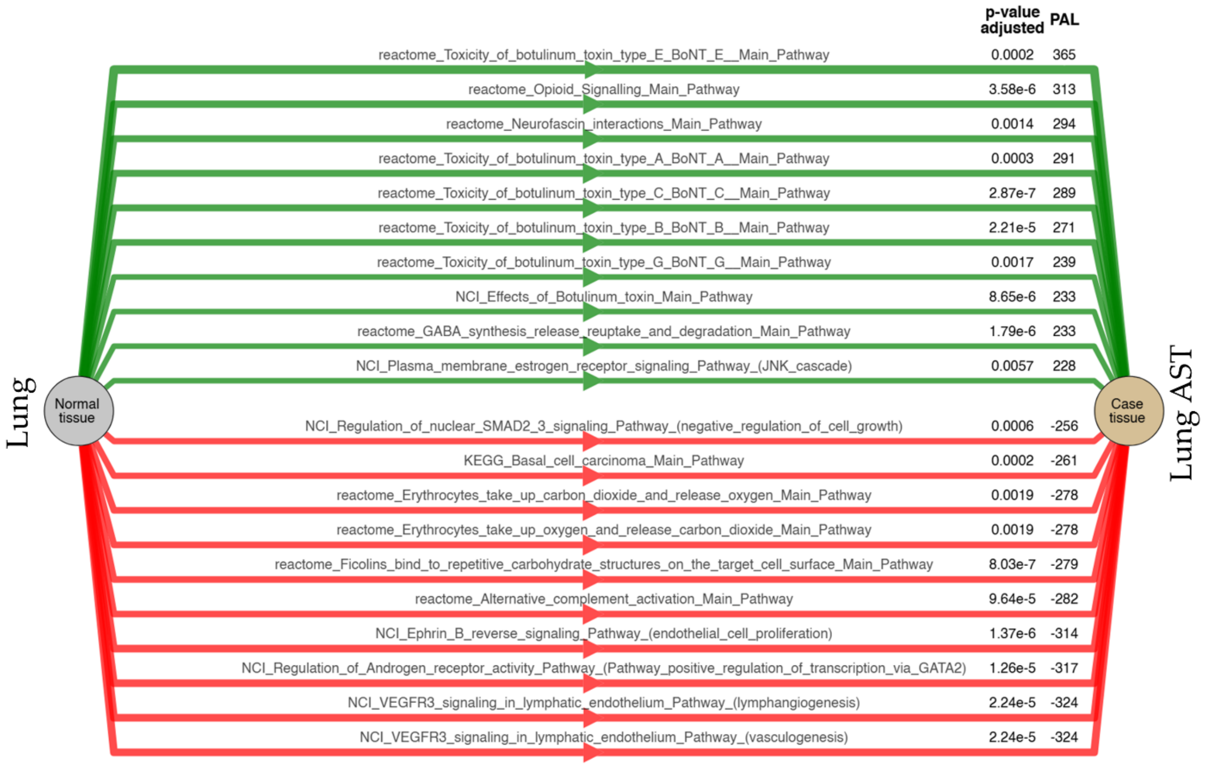

Figure S6. Top 10 upregulated and downregulated molecular pathways in lung ASTs compared to normal lung tissue. Pathway Activation Level (PAL) values for the top differentially activated molecular pathways are shown. Pathways with increased activation are highlighted in green and have positive PAL values, while inhibited pathways are highlighted in red with negative PAL values. FDR-adjusted p-values indicate statistical significance of the differential regulation of pathways.

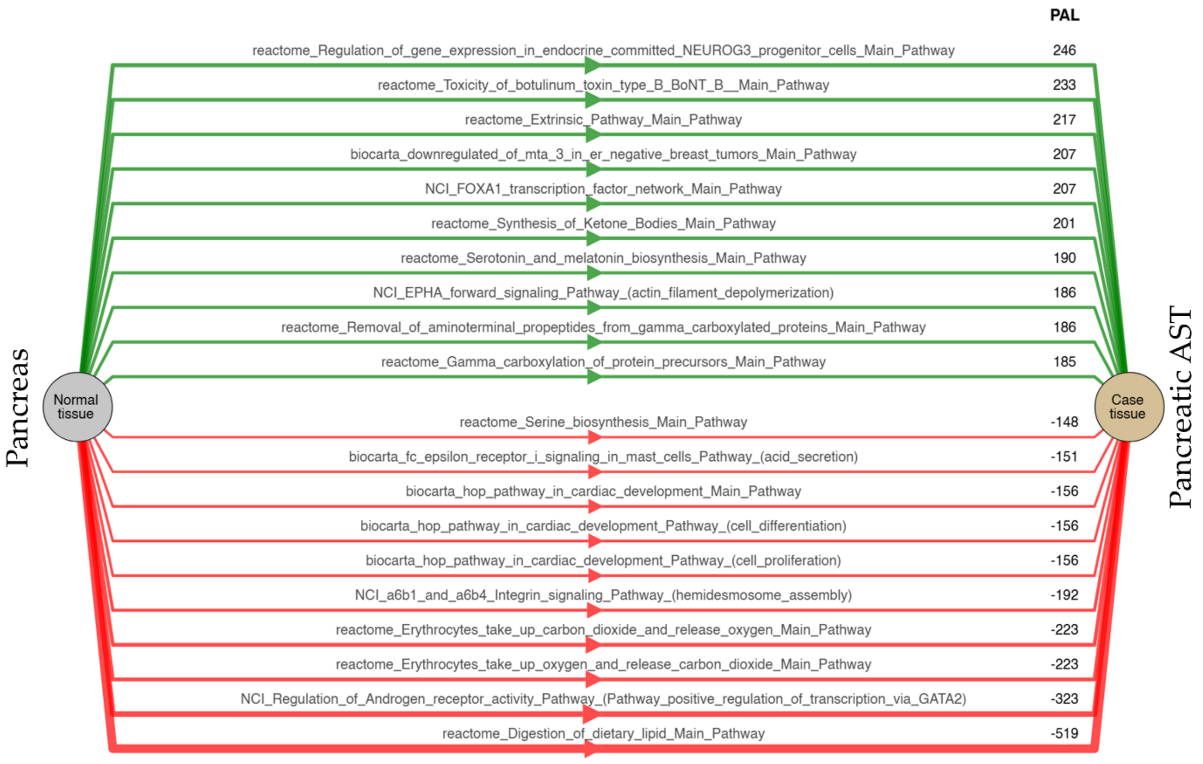

Figure S7. Top 10 upregulated and downregulated molecular pathways in pancreatic AST compared to normal pancreas tissue. Pathway Activation Level (PAL) values for the top differentially activated molecular pathways are shown. Pathways with increased activation are highlighted in green and have positive PAL values, while inhibited pathways are highlighted in red with negative PAL values.

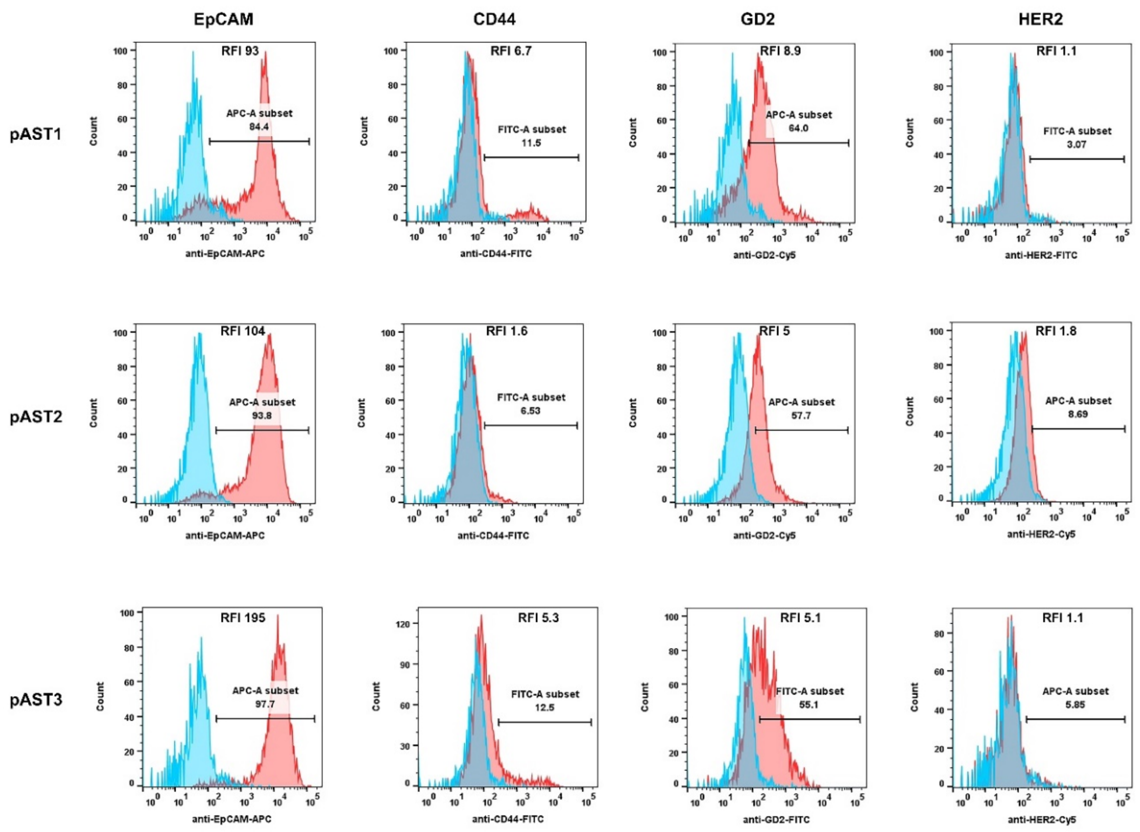

Figure S8. Flow cytometry analysis of CD44, EpCAM, GD2 and HER2 expression by cells from biopsy of patient with pituitary ASTs. Blue peak – autofluorescence of the unstained cells, red peak – fluorescence of the antibody-stained cells. Marker indicates fraction of positively stained cells (%).
